# New Cembrane-Type Diterpenoids from the South China Sea Soft Coral *Sarcophyton ehrenbergi*

**DOI:** 10.3390/molecules21050587

**Published:** 2016-05-04

**Authors:** Gui-Hua Tang, Zhang-Hua Sun, Yi-Hong Zou, Sheng Yin

**Affiliations:** 1 School of Pharmaceutical Sciences, Sun Yat-sen University, Guangzhou 510006, China; tanggh5@mail.sysu.edu.cn (G.-H.T.); sunzh@gdim.cn (Z.-H.S.); zouyh5@mail2.sysu.edu.cn (Y.-H.Z.); 2 State Key Laboratory of Applied Microbiology Southern China, Guangdong Institute of Microbiology, Guangzhou 510070, China

**Keywords:** *Sarcophytonehrenbergi*, cembranoids, marine natural product

## Abstract

Chemical investigation on the soft coral *Sarcophyton* *ehrenbergi* collected from the Xisha Islands of the South China Sea have led to the isolation of eight cembranoids including five new ones, sarcophytonoxides A–E (**1**–**5**). The structures of new cembranoids (**1**–**5**) were determined by spectroscopic analysis and comparison of the NMR data with those of related analogues. The cytotoxicities of compounds **1**–**8** against human ovarian cancer cell line A2780 were also evaluated.

## 1. Introduction

**Figure 1:**
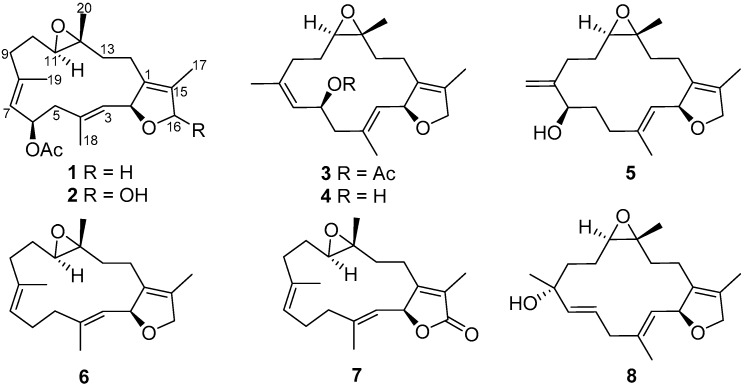
The structures of compounds **1**–**8** isolated from *Sarcophyton ehrenbergi*.

Our previous chemical investigations on soft corals belonging to the genus *Sarcophyton* (Alcyoniidae), collected off the waters of the South China Sea, have obtained a series of marine natural products, including prostaglandin derivatives [1], sarsolenane-, capnosane- and cembrane-type diterpenoids [2,3,4]. These chemical components, especially cembranes, possessed various bioactivities such as anti-protozoal [5], cytotoxic [6,7], antiviral [8], and anti-inflammatory [9,10] properties, which have motivated natural product researchers to search for potential drug leads. In the course of our ongoing work, five new cembrane-type dierpenoids, sarcophytonoxides A–E (**1**–**5**), together with three known ones (Figure 1), were isolated from *Sarcophyton ehrenbergi* (Figure S1), a soft coral sample collected from North Reef (Beijiao) in the Xisha Islands of the South China Sea. In this paper, we focus on the isolation, structure elucidation, and cytotoxic activities of compounds **1**–**8**.

## 2. Results and Discussion

The frozen bodies of *S. ehrenbergi* were extracted by acetone to yield a black residue, which was suspended in water and successively partitioned with petroleum ether (PE) and EtOAc. The EtOAc partition was chromatographed over Sephadex LH-20, silica column, and semipreparative HPLC to obtain eight cembranoids (**1**–**8**) (Figure 1).

A sodiated molecular ion peak at *m*/*z* 383.2206 [M + Na]^+^ (calcd for C_22_H_32_O_4_Na^+^, 383.2193) observed in the HRESIMS in conjunction with the ^13^C-NMR data corresponded to a molecular formula of C_22_H_32_O_4_ with seven degrees of unsaturation for sarcophytonoxide A (**1**). The IR spectrum showed bands at 1729 and 1665 cm^−1^ for carbonyl group and double bonds absorptions. The resonances in the ^1^H-NMR spectrum (Table 1) for the five methyls (δ_H_ 1.97, 1.84, 1.67, 1.65, and 1.26 (each 3H, s)) were all singlet signals, indicative of quaternary methyls. The resonances at δ_H_ 2.31 (dd, *J* = 11.3 and 2.3 Hz) and δ_C_ 61.8 (CH) and 61.4 (C) suggested the presence of an epoxy. The ^13^C-NMR spectrum (Table 2) of **1** showed 22 carbon signals, which were classified from the DEPT and HSQC spectra into five methyls (an acetyl methyl), six methylenes (one oxygenated), five methines (two olefinic and three oxygenated ones), and six quaternary carbons (one oxygenated quaternary carbon, one ester carbonyl carbon, and four olefinic quaternary ones). It was obvious from comparison of the aforementioned data with those of (2S*,11R*,12R*)-isosarcophytoxide (**6**) [11] and from analysis of the 2D NMR correlations of **1** that sarcophytonoxide A was a cembrane diterpenoid characterized by an epoxide, a dihydrofuran, three olefin bonds, and an acetyl group.

**Figure 2:**
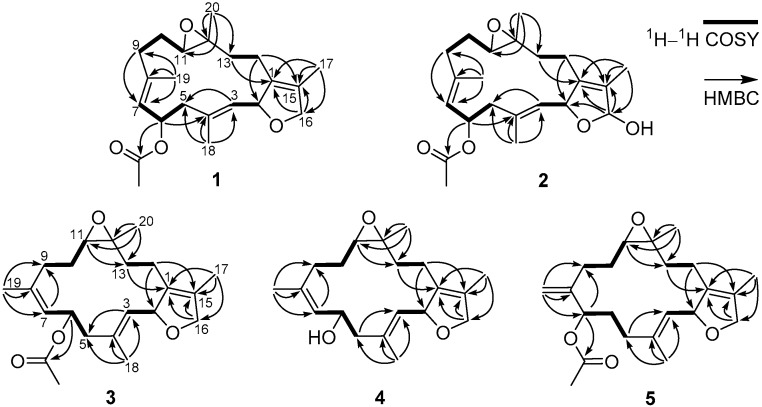
The ^1^H-^1^H COSY and selected HMBC correlations of compounds **1**–**5**.

**Figure 3:**
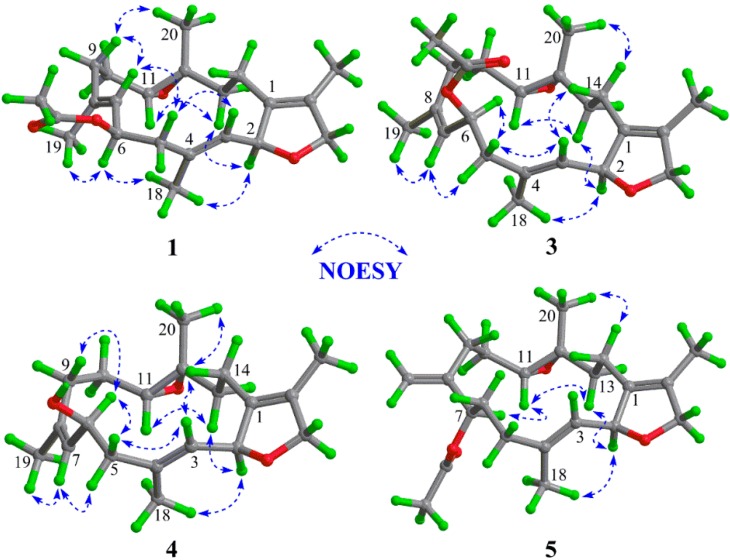
Key NOESY correlations of compounds **1** and **3**–**5**.

The ^1^H-^1^H COSY spectrum exhibited four partial structures (Figure 2), C-2 to C-3, C-5 to C-7, C-9 to C-11, and C-13 to C-14. Based on the HMBC correlations (Figure 2) of H_3_-18 to C-3, C-4, and C-5, H_3_-19 to C-7, C-8, and C-9, H_3_-20 to C-11, C-12, and C-13, H_3_-17 to C-1, C-15, and C-16, and H_2_-14 to C-2 and C-15, these fragments and quaternary carbons were connected to form a carbon skeleton of cembrane-type diterpenoid with 14-membered macrocycle fused with a dihydrofuran at C-1 and C-2 and with an epoxy at C-11 and C-12, and substituted by three symmetrically disposed methyl groups at positions C-4, C-8, and C-12. The acetyl group was located at C-6 by the HMBC correlation of H-6 (δ_H_ 5.79) to the acetyl carbonyl carbon (δ_C_ 170.2). The geometry of two trisubstituted-double bonds and the configurations at four chiral centers were assigned by analysis of its NOESY data. The *E*-configurations for both Δ^3,4^ and Δ ^7,8^ were determined by the NOESY correlations of H-2/H_3_-18 and H-6/H_3_-19, respectively. NOESY correlations shown in Figure 3 assigned H-2, H-6, and H-11 as α-oriented, while CH_3_-20 was the β-orientation. Thus, in a relative sense, C-2, C-6, C-11, and C-12 in sarcophytonoxide A (**1**) were assigned the S*, R*, R*, and R* configurations, respectively.

Compound **2** was assigned the molecular formula C_22_H_32_O_5_, 16 mass units more than that of **1**, by the analysis of the HRESIMS and NMR data. The ^1^H- and ^13^C-NMR data (Table 1 and Table 2) of **2** closely matched those of **1**; however, **2** contained resonances for a hemiacetal group (δ_H_ 5.78 (1H, s) and δ_C_ 114.8 (CH)) instead of the signals of the oxygenated methylene group present in **1**. Both Δ^3,4^ and Δ^7,8^ were assigned as *E*-configuration, and the configurations at C-2, C-6, C-11, and C-12 were determined to be the same as **1** by analysis of the NOESY spectrum of **2**. Thus, the structure of **2** was deduced as shown and named sarcophytonoxide B.

The same molecular formula as that of **1**, C_22_H_32_O_4_, was assigned to sarcophytonoxide C (**3**) based on the ion peak at *m*/*z* 383.2204 [M + Na]^+^ (calcd for C_22_H_32_O_4_Na^+^, 383.2193) and the ^13^C-NMR data. Comparison of the ^1^H- and ^13^C-NMR data of **3** with those of **1** (see Table 1 and Table 2) showed that **3** was very similar to sarcophytonoxide A (**1**). The main differences were the carbon chemical shifts of C-19 (δ_C_ 22.7 in **3**, δ_C_ 15.1 in **1**, Δδ = +7.6 ppm), C-6 (δ_C_ 72.4 in **3**, δ_C_ 68.3 in **1**, Δδ = +4.1 ppm), C-7 (δ_C_ 131.3 in **3**, δ_C_ 125.1 in **1**, Δδ = +6.0 ppm), and C-9 (δ_C_ 29.2 in **3**, δ_C_ 37.2 in **1**, Δδ = −8.0 ppm), which may be due to the configuration of 7,8-double bond or the C-6 chiral center. The key NOESY cross-peak of H-7/H_3_-19 confirmed the *Z*-configuration of Δ^7,8^. The *E*-configuration for Δ^3,4^ was determined by the NOESY correlation of H-2/H_3_-18. Moreover, the configurations of C-2, C-6, C-11, and C-12 were deduced to be the S*, S*, R*, and R*, respectively, by analysis of its NOESY correlations (Figure 3). Therefore, the structure of **3** was deduced as shown and named sarcophytonoxide C.

The HRESIMS of compound **4** showed a sodiated molecular ion peak at *m*/*z* 341.2102 [M + Na]^+^ (calcd for C_20_H_30_O_3_Na^+^, 341.2087), corresponding to a molecular formula of C_20_H_30_O_3_. Detail analysis of its 1D NMR (see Table 1 and Table 2) and MS data with those of **3** suggested that the acetyl group in **3** was replaced by a hydroxyl group in **4**, which indicated that **4** was a deacetylated derivative of **3**. On the basis of the NOESY correlations (Figure 3), the relative configurations of **4** were determined as the same as **3**. Hence, the structure of **4** was well established and named as sarcophytonoxide D.

Sarcophytonoxide E (**5**) had the molecular formula C_22_H_32_O_4_ with seven degrees of unsaturation based on the [M + Na]^+^ at *m*/*z* 383.2208 (calcd for C_22_H_32_O_4_Na^+^, 383.2193) in its HREIMS. The 1D NMR data (Table 1 and Table 2) displayed signals for four methyl groups including an acetyl methyl, an exocyclic double bond (δ_H_ 5.11 and 5.01 (each 1H, s); δ_C_ 151.3 (C) and 112.7 (CH_2_)), a trisubstituted double bond, a tetrasubstituted double bond, an epoxy group (δ_H_ 2.49 (1H, dd, *J* = 8.8 and 2.8 Hz); δ_C_ 63.2 (CH) and 61.8 (C)), an oxygenated methyl (δ_H_ 4.40 (2H, m); δ_C_ 78.5 (CH_2_)), two oxygenated methines, and six methylenes. The collective data implied that **5** was homologous to sarcophytonoxides A–D (**1**–**4**). Comparison of the 1D NMR data of **5** with those of sarcophytonoxide C showed that the main differences were the presence of an exocyclic double bond and the location of the acetyl group. Analysis of its 2D NMR data (Figure 2) confirmed the exocyclic double bond at C-9 and C-19, and the acetyl group linked at C-7. An *E*-configuration for the 3,4-double bond was determined by the NOESY correlation of H-2/H_3_-18. The key NOESY correlations of H-2/ H-13α, H-13α/H-11, H-11/H-7, and H_3_-20/H-14 (Figure 3) assigned the S*, R*, R*, and R* configurations for C-2, C-7, C-11, and C-12, respectively. Thus, the structure of **5** was established as shown, and named sarcophytonoxide E.

The known compounds, (2S*,11R*,12R*)-isosarcophytoxide (**6**) [11], (+)-isosarcophine (**7**) [12], and 8-hydroxyisosarcophytoxide-6-ene (**8**) [12], were identified by comparison of their NMR and MS data with those in the literature.

All of the cembranoids from *S. ehrenbergi* were evaluated for their inhibitory activities against human ovarian cancer cell line A2780 using MTT method [13]. The results showed that all compounds were inactive (IC_50_ > 25 µM) against A2780 cells.

## 3. Materials and Methods

### 3.1. General

Optical rotations were measured on a Perkin-Elmer 341 polarimeter (Perkin-Elmer, Waltham, MA, USA). IR spectra were recorded on a Bruker Tensor 37 infrared spectrophotometer (Bruker, Karlsruhe, Germany). 1D and 2D NMR spectra (400 MHz for ^1^H and 100 MHz for ^13^C, respectively) were measured on a Bruker AM-400 spectrometer (Bruker) at 25 °C. ESIMS spectra were performed on a Finnigan LCQ Deca instrument (Bruker), and HRESIMS was measured on a Waters Micromass Q-TOF spectrometer (Waters, Milford, MA, USA). HPLC was performed with a YMC-pack ODS-A column (250 mm × 10 mm, S-5 µm, 12 nm) (YMC, Tokyo, Japan) or a Phenomenex Lux cellulose-2 chiral column (250 mm × 10 mm, 5 µm) (Phenomenex, Torrance, CA, USA) under Shimadzu LC-20 AT equipped with a SPD-M20A PDA detector (Shimadzu, Kyoto, Japan). Column chromatography (CC) was performed on silica gel (300–400 mesh, Qingdao Haiyang Chemical Co., Ltd., Qingdao, China), C_18_ reversed-phase silica gel (12 nm, S-50 µm, YMC Co., Ltd., Tokyo, Japan), and Sephadex LH-20 gel (Amersham Biosciences, Uppsala, Sweden) For RP-HPLC and CC, the analytical grade solvents (Guangzhou Chemical Reagents Company, Ltd., Guangzhou, China) were employed.

### 3.2. Animal Material

The soft coral *S. ehrenbergi* (specimen No. RSH201410) was collected by hand, using scuba from North Reef (Beijiao) in the Xisha Islands of the South China Sea, in October 2014, at a water depth of 4–5 m, and stored in a freezer until extraction. A voucher specimen identified by Cheng-Qi Fan, East China Sea Fisheries Research Institute, Chinese Academy of Fishery Sciences, was deposited at the School of Pharmaceutical Sciences, Sun Yat-sen University.

### 3.3. Extraction and Isolation

The frozen bodies of *S. ehrenbergi* (600 g, wet weight) were chopped and exhaustively extracted with acetone (1 L × 3) at room temperature. The combined acetone extracts were concentrated *in vacuo* to a black residue (7 g), which was suspended in water (120 mL) and then partitioned with petroleum ether (3 × 200 mL) and EtOAc (3 × 200 mL), respectively. The dried EtOAc extract (1.2 g) was chromatographed over Sephadex LH-20 eluted with EtOH to give three fractions (Fr. I–III). Fr. I (827 mg) was subjected to gravity chromatography in a silica gel column using PE/acetone gradient (80:1→3:1) separated into seven fractions (Fr. Ia–Ig). Fr. Ib (297 mg) was purified on a semipreparative reversed-phase (RP) HPLC system equipped with a YMC column (MeCN/H_2_O, 70:30, 3 mL/min) to give **7** (33 mg, *t*_R_ 8.7 min) and **6** (131 mg, *t*_R_ 14.5 min). Fr. Id (226 mg) was subjected to RP-HPLC (MeCN/H_2_O, 70:30, 3 mL/min) to afford **5** (22 mg, *t*_R_ 10.8 min), **8** (17 mg, *t*_R_ 13.2 min), and a mixture (*t*_R_ 17.8 min). Further purification of the mixture (105 mg) on HPLC equipped with a chiral-phase column (Phenomenex Lux, cellulose-2, MeCN/H_2_O, 75:25, 3 mL/min) yielded **3** (24 mg, *t*_R_ 15.0 min) and **1** (28 mg, *t*_R_ 18.6 min). Fr. Ie (68 mg) was subjected to further purification by RP-HPLC (MeCN/H_2_O, 60:40, 3 mL/min) to give **4** (13 mg, *t*_R_ 11.9 min). Fr. If (46 mg) was chromatographed over RP-HPLC (MeCN/H_2_O, 60:40, 3 mL/min) to give **2** (11 mg, *t*_R_ 14.7 min).

### 3.4. Bioactivity Assays

The isolated cembrane-type diterpenoids were tested *in vitro* for their cytotoxicity against human ovarian cancer cell line A2780 using the MTT assay [13]. In brief, A2780 cells in the log phase of their growth cycle were seeded in 96-well plates with 5.0 × 10^3^ cells/well in a volume of 100 µL. The cells were grown in a humidified 5% CO_2_ atmosphere at 37 °C overnight. Then the test compounds in complete growth medium (100 µL) were added to the wells in triplicate. After incubation for 48 h at 37 °C, a 20 μL aliquot of MTT solution (5 mg/mL) was added to each well. Incubation was continued for another 3 h, the supernatant was removed, and 100 µL of dimethyl sulfoxide (DMSO) was added. Finally, absorbance in each well was measured at 570 nm on a TECAN infinite M200 pro multimode reader. The 50% inhibitory concentration (IC_50_) was obtained by nonlinear regression analysis of logistic curves.

*Sarcophytonoxide A* (**1**)*: * Colorless oil; ${\left[\alpha \right]}_{D}^{25}$ −36.8 (*c* 0.5, MeOH); IR (KBr) ν_max_ 1729, 1665, 1440, 1371, 1241, 1039, 1017, 948 cm^−1^; ^1^H- and ^13^C-NMR data, see Table 1 and Table 2; ESIMS *m*/*z* 383 [M + Na]^+^, HRESIMS *m*/*z* 383.2206 [M + Na]^+^ (calcd for C_22_H_32_O_4_Na^+^, 383.2193).

*Sarcophytonoxide B* (**2**): Colorless oil; ${\left[\alpha \right]}_{D}^{25}$ −12.5 (*c* 0.5, MeOH); IR (KBr) ν_max_ 3453, 1731, 1668, 1439, 1373, 1244, 1020, 950 cm^−1^; ^1^H- and ^13^C-NMR data, see Table 1 and Table 2; ESIMS *m*/*z* 399 [M + Na]^+^, HRESIMS *m*/*z* 399.2158 [M + Na]^+^ (calcd for C_22_H_32_O_5_Na^+^, 399.2142).

*Sarcophytonoxide C* (**3**): Colorless oil; ${\left[\alpha \right]}_{D}^{25}$ −50.7 (*c* 0.5, MeOH); IR (KBr) ν_max_ 1730, 1663, 1445, 1371, 1237, 1040, 942 cm^−1^; ^1^H- and ^13^C-NMR data, see Table 1 and Table 2; ESIMS *m*/*z* 383 [M + Na]^+^, HRESIMS *m*/*z* 383.2204 [M + Na]^+^ (calcd for C_22_H_32_O_4_Na^+^, 383.2193).

*Sarcophytonoxide D* (**4**): Colorless oil; ${\left[\alpha \right]}_{D}^{25}$ −28.0 (*c* 0.5, MeOH); IR (KBr) ν_max_ 3455, 1733, 1699, 1662, 1448, 1384, 1249, 1182, 1038, 980, 938 cm^−1^; ^1^H- and ^13^C-NMR data, see Table 1 and Table 2; ESIMS *m*/*z* 341 [M + Na]^+^, HRESIMS *m*/*z* 341.2102 [M + Na]^+^ (calcd for C_20_H_30_O_3_Na^+^, 341.2087).

*Sarcophytonoxide E* (**5**): Colorless oil; ${\left[\alpha \right]}_{D}^{25}$ −19.6 (*c* 0.5, MeOH); IR (KBr) ν_max_ 1735, 1646, 1448, 1372, 1237, 1038 cm^−1^; ^1^H- and ^13^C-NMR data, see Table 1 and Table 2; ESIMS *m*/*z* 383 [M + Na]^+^, HRESIMS *m*/*z* 383.2208 [M + Na]^+^ (calcd for C_22_H_32_O_4_Na^+^, 383.2193).

## 4. Conclusions

Soft corals from the South China Sea belonging to the genus *Sarcophyton*, a number of cembrane-type diterpenoids, were isolated by our research group. In our continuing search for marine natural product from the soft corals of *Sarcophyton* sp., five new cembranoids, sarcophytonoxides A–E (**1**–**5**), along with three known ones, (2S*,11R*,12R*)-isosarcophytoxide (**6**), (+)-isosarcophine (**7**), and 8-hydroxyisosarcophytoxide-6-ene (**8**), were obtained from the soft coral *S.* *ehrenbergi* collected from the Xisha Islands of the South China Sea. The cytotoxicity assay results showed that all cembranoids were inactive (IC_50_ > 25 µM) against A2780 cells.

## Figures and Tables

**Table 1:** ^1^H-NMR spectroscopic data of **1**–**5** in CD_3_COCD_3_ (400 MHz, δ in ppm, *J* in Hz).

Position	1	2	3	4	5
2	5.36, m	5.28, d (10.4)	5.35, dd (10.4, 2.9)	5.34, brs	5.40, m
3	5.08, d (10.1)	5.13, d (10.4)	5.17, d (10.0)	5.10, d (9.9)	5.00, d (9.6)
5	2.62, dd (12.5, 5.2); 2.22, dd (12.5, 11.1)	2.64, dd (12.4, 5.0); 2.23, t (11.8)	2.47, dd (11.9, 3.0); 2.27, t (11.4)	2.50, dd (12.0, 3.1); 2.12, t (11.1)	2.17, m
6	5.79, m	5.80, m	5.32, dd (9.9, 2.9)	4.19, t (8.2)	2.02, m; 1.77, m
7	5.22, d (8.9)	5.23, d (9.6)	5.49, d (9.6)	5.46, d (8.9)	5.12, dd (9.7, 1.4)
9	2.34, m; 2.09, m	2.36, m; 2.12, m	2.59, td (14.2, 3.0); 1.98, m	2.59, td (14.4, 2.6); 2.01, m	2.32, m
10	2.10, m; 1.26, m	2.11, m; 1.28, m	2.01, m; 1.42, m	2.00, m; 1.41, m	1.87, m; 1.57, m
11	2.31, dd (11.3, 2.3)	2.32, dd (11.1, 1.7)	2.36, dd (10.5, 1.8)	2.37, dd (10.0, 1.5)	2.49, dd (8.8, 2.8)
13	1.80, m; 0.89, t (12.2)	1.86, m; 0.95, m	1.83, m; 1.12, t (7.0)	1.78, m; 1.06, m	1.90, m; 1.09, td (13.0, 3.0)
14	2.40, dd (12.6, 5.3); 1.74, m	2.45, m; 1.80, m	2.12, m; 1.81, m	2.15, m; 1.77, m	2.28, m; 2.12, m
16	4.40, qd (11.7, 4.2)	5.78, s	4.40, qd (11.7, 4.3)	4.40, qd (11.6, 5.0)	4.40, m
17	1.67, s	1.70, s	1.66, s	1.66, s	1.68, s
18	1.65, s	1.68, s	1.76, s	1.73, s	1.78, s
19	1.84, s	1.85, s	1.85, s	1.81, s	5.11, s; 5.01, s
20	1.26, s	1.27, s	1.27, s	1.26, s	1.26, s
OAc	1.97, s	1.97, s	1.95, s		2.08, s

**Table 2:** ^13^C-NMR spectroscopic data of **1**–**5** in CD_3_COCD_3_ (100 MHz, δ in ppm, *J* in Hz).

Position	1	2	3	4	5
1	133.4, C	140.8, C	133.2, C	133.5, C	133.9, C
2	83.5, CH	82.1, CH	84.4, CH	84.5, CH	84.4, CH
3	130.3, CH	129.7, CH	128.2, CH	130.0, CH	129.2, CH
4	135.4, C	136.1, C	135.4, C	136.9, C	137.4, C
5	45.4, CH_2_	45.3, CH_2_	46.9, CH_2_	50.5, CH_2_	36.6, CH_2_
6	68.3, CH	68.3, CH	72.4, CH	70.4, CH	30.7, CH_2_
7	125.1, CH	125.1, CH	131.3, CH	133.3, CH	72.8, CH
8	142.3, C	142.4, C	141.2, C	137.4, C	151.3, C
9	37.2, CH_2_	37.2, CH_2_	29.2, CH_2_	28.7, CH_2_	32.6, CH_2_
10	24.5, CH_2_	24.5, CH_2_	24.4, CH_2_	24.4, CH_2_	31.0, CH_2_
11	61.8, CH	61.7, CH	60.4, CH	60.7, CH	63.2, CH
12	61.4, C	61.4, C	61.3, C	61.3, C	61.8, C
13	38.1, CH_2_	38.1, CH_2_	36.9, CH_2_	37.2, CH_2_	36.8, CH_2_
14	22.9, CH_2_	23.2, CH_2_	21.9, CH_2_	22.1, CH_2_	21.9, CH_2_
15	129.3, C	126.5, C	129.5, C	129.3, C	128.9, C
16	78.6, CH_2_	114.8, CH	78.7, CH_2_	78.6, CH_2_	78.5, CH_2_
17	9.9, CH_3_	10.0, CH_3_	10.0, CH_3_	10.0, CH_3_	10.2, CH_3_
18	15.4, CH_3_	15.4, CH_3_	18.2, CH_3_	18.3, CH_3_	15.9, CH_3_
19	15.1, CH_3_	15.1, CH_3_	22.7, CH_3_	22.7, CH_3_	112.7, CH_2_
20	15.9, CH_3_	15.9, CH_3_	17.0, CH_3_	16.9, CH_3_	16.8, CH_3_
OAc	170.2, C	170.3, C	170.0, C		170.7, C
	21.2, CH_3_	21.2, CH_3_	21.2, CH_3_		21.0, CH_3_

## Supplementary Materials

Supplementary materials can be accessed at: http://www.mdpi.com/1420-3049/21/5/587/s1.
