# Supplementary material for: New Cembrane-Type Diterpenoids from the South China Sea Soft Coral Sarcophyton ehrenbergi
Source: Molecules. 2016 May 4;21(5):587. doi: 10.3390/molecules21050587 (PMC6273003; doi:10.3390/molecules21050587)
Supplement: Supplementary file 1 [file molecules-21-00587-s001.pdf]

Supplementary Materials: New Cembrane-Type Diterpenoids from the South China Sea Soft Coral *Sarcophyton ehrenbergi*

Gui-Hua Tang, Zhang-Hua Sun, Yi-Hong Zou and Sheng Yin

Contents:

Figure S1. The South China Sea soft coral *Sarcophyton ehrenbergi*

Figure S2. ^{1}H-NMR spectrum of compound **1**

Figure S3. DEPT spectra of compound **1**

Figure S4. HSQC spectrum of compound **1**

Figure S5. ^{1}H–^{1}H COSY spectrum of compound **1**

Figure S6. HMBC spectrum of compound **1**

Figure S7. NOESY spectrum of compound **1**

Figure S8. ^{1}H-NMR spectrum of compound **2**

Figure S9. DEPT spectra of compound **2**

Figure S10. HSQC spectrum of compound **2**

Figure S11. ^{1}H–^{1}H COSY spectrum of compound **2**

Figure S12. HMBC spectrum of compound **2**

Figure S13. NOESY spectrum of compound **2**

Figure S14. ^{1}H-NMR spectrum of compound **3**

Figure S15. DEPT spectra of compound **3**

Figure S16. HSQC spectrum of compound **3**

Figure S17. ^{1}H–^{1}H COSY spectrum of compound **3**

Figure S18. HMBC spectrum of compound **3**

Figure S19. NOESY spectrum of compound **3**

Figure S20. ^{1}H-NMR spectrum of compound **4**

Figure S21. DEPT spectra of compound **4**

Figure S22. HSQC spectrum of compound **4**

Figure S23. ^{1}H–^{1}H COSY spectrum of compound **4**

Figure S24. HMBC spectrum of compound **4**

Figure S25. NOESY spectrum of compound **4**

Figure S26. ^{1}H-NMR spectrum of compound **5**

Figure S27. DEPT spectra of compound **5**

Figure S28. HSQC spectrum of compound **5**

Figure S29. ^{1}H–^{1}H COSY spectrum of compound **5**

Figure S30. HMBC spectrum of compound **5**

Figure S31. NOESY spectrum of compound **5**

Figure S32. ^{1}H-NMR spectrum of compound **6**

Figure S33. DEPT spectra of compound **6**

Figure S34. ^{1}H-NMR spectrum of compound **7**

Figure S35. DEPT spectra of compound **7**

Figure S36. ^{1}H-NMR spectrum of compound **8**

Figure S37. DEPT spectra of compound **8**

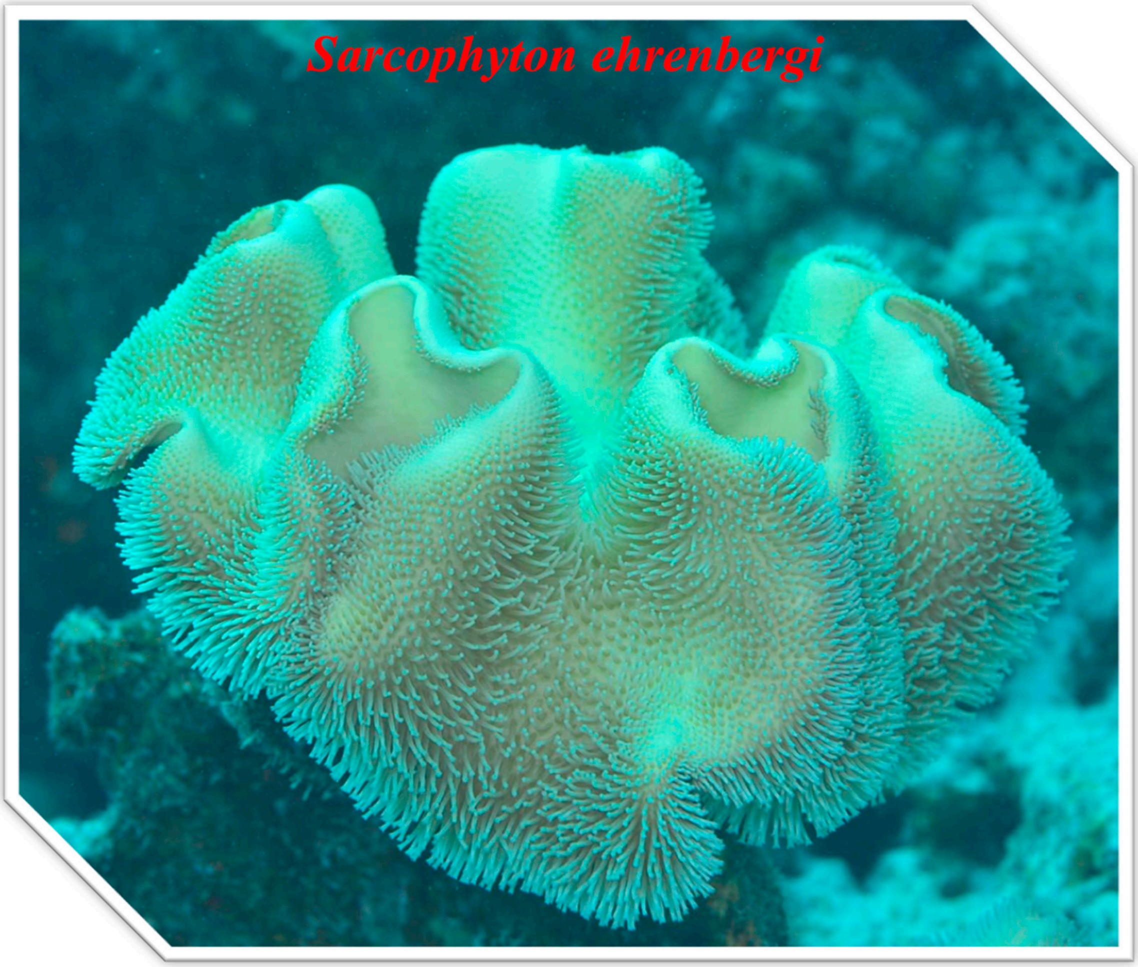

Figure S1. The South China Sea soft coral *Sarcophyton ehrenbergi*.

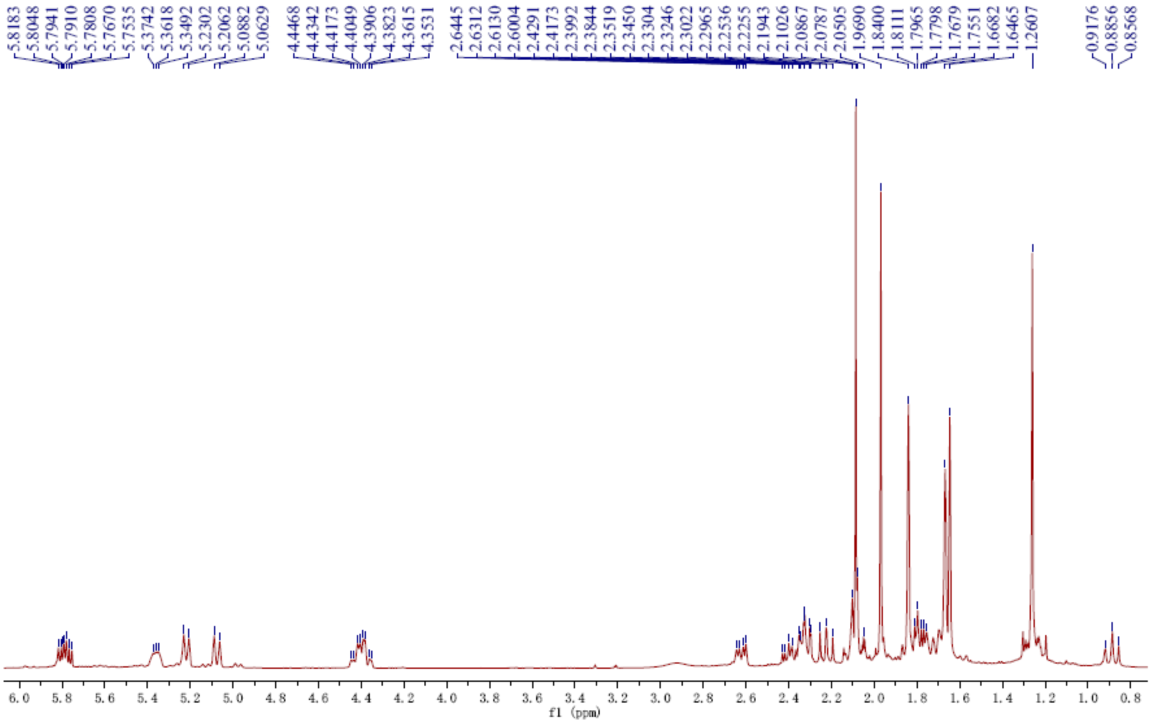

Figure S2. ^{1}H-NMR spectrum of compound **1**.

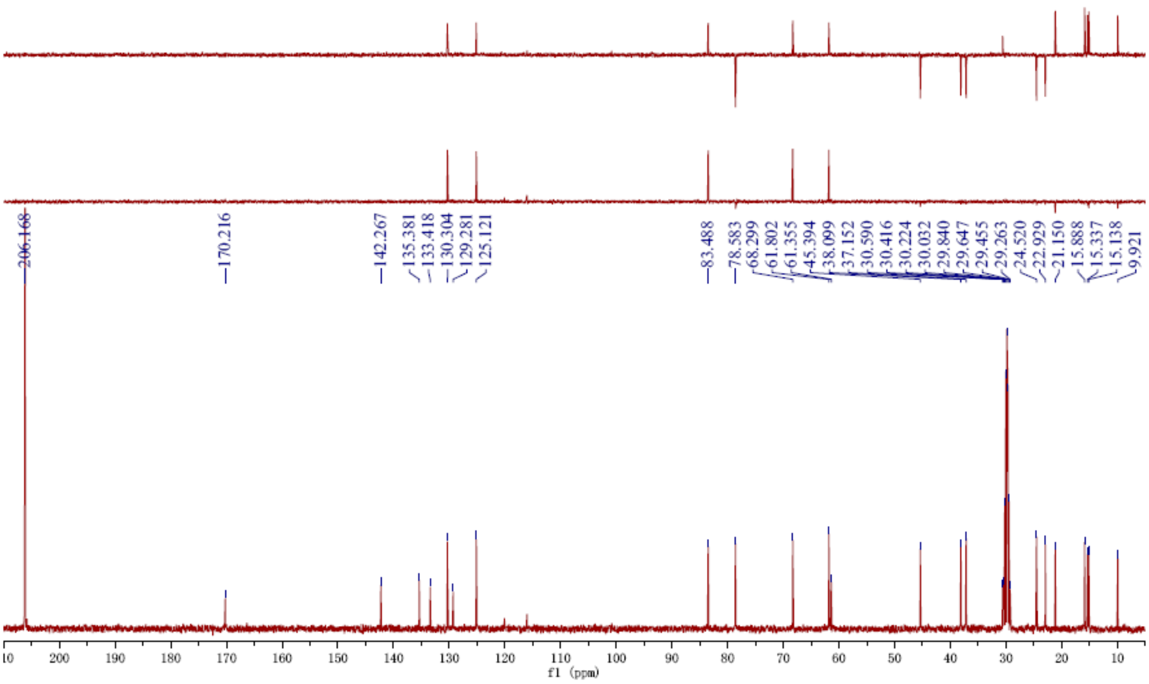

Figure S3. DEPT spectra of compound **1**.

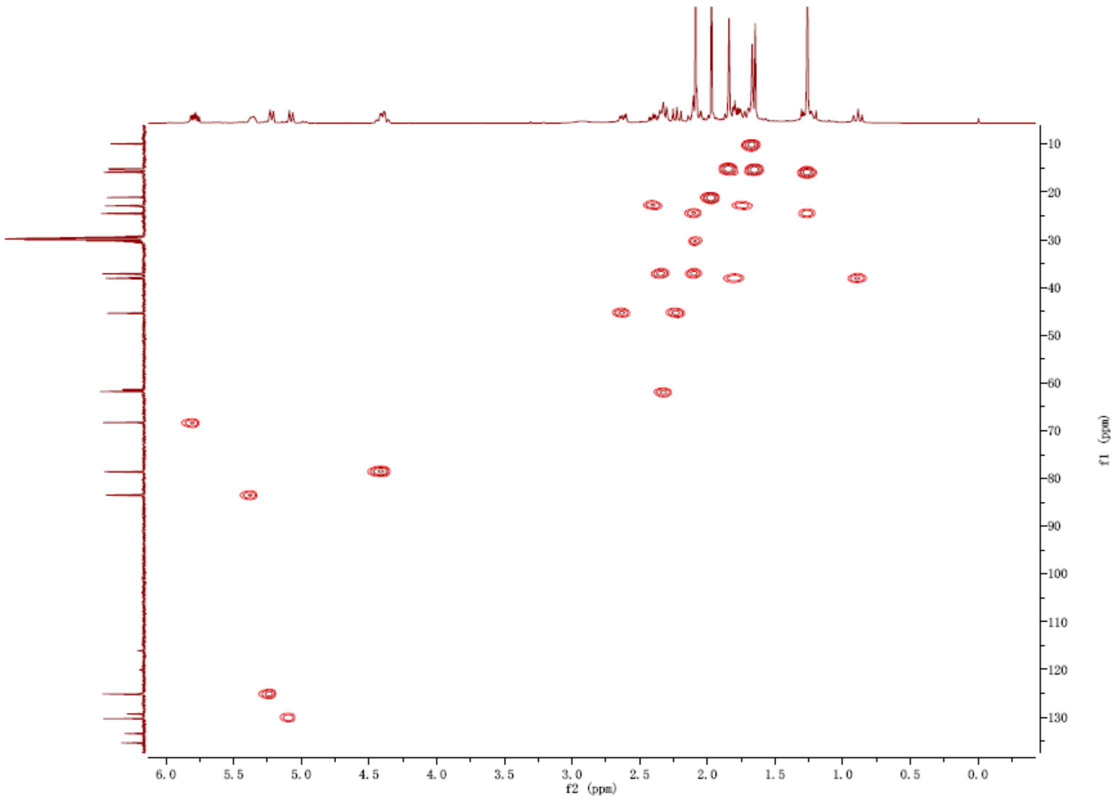

Figure S4. HSQC spectrum of compound **1**.

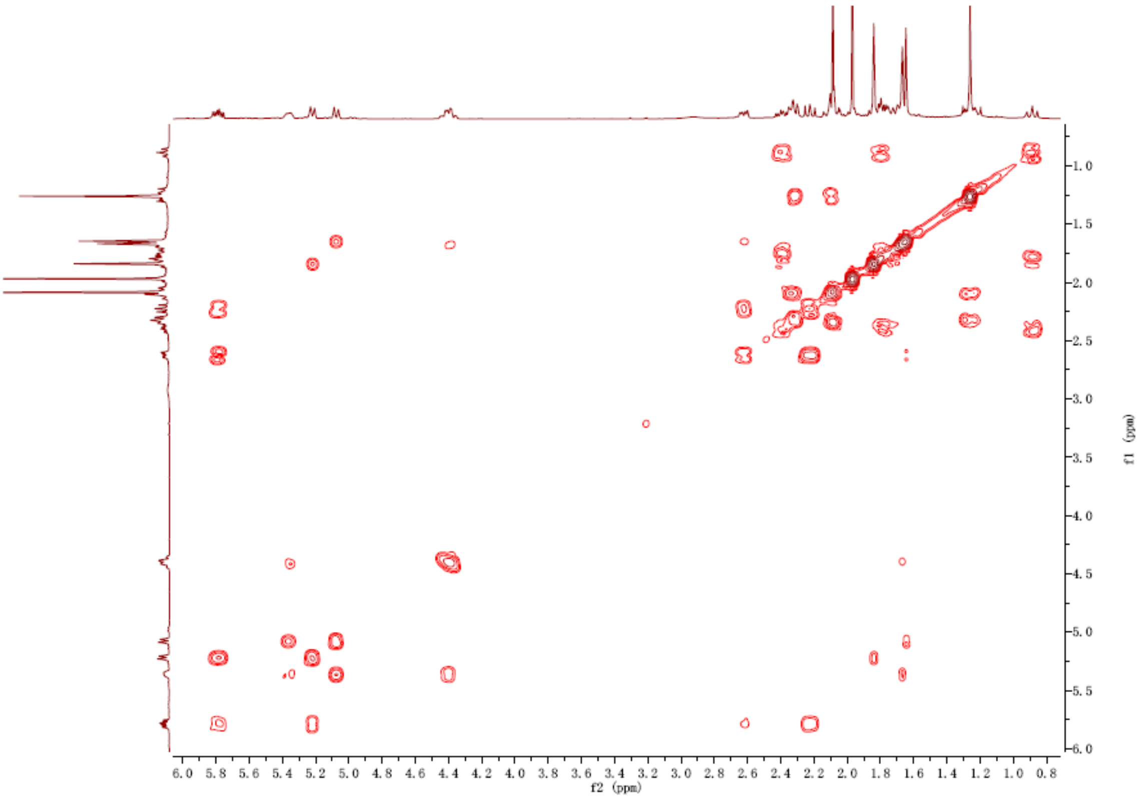

Figure S5. ^{1}H–^{1}H COSY spectrum of compound **1**.

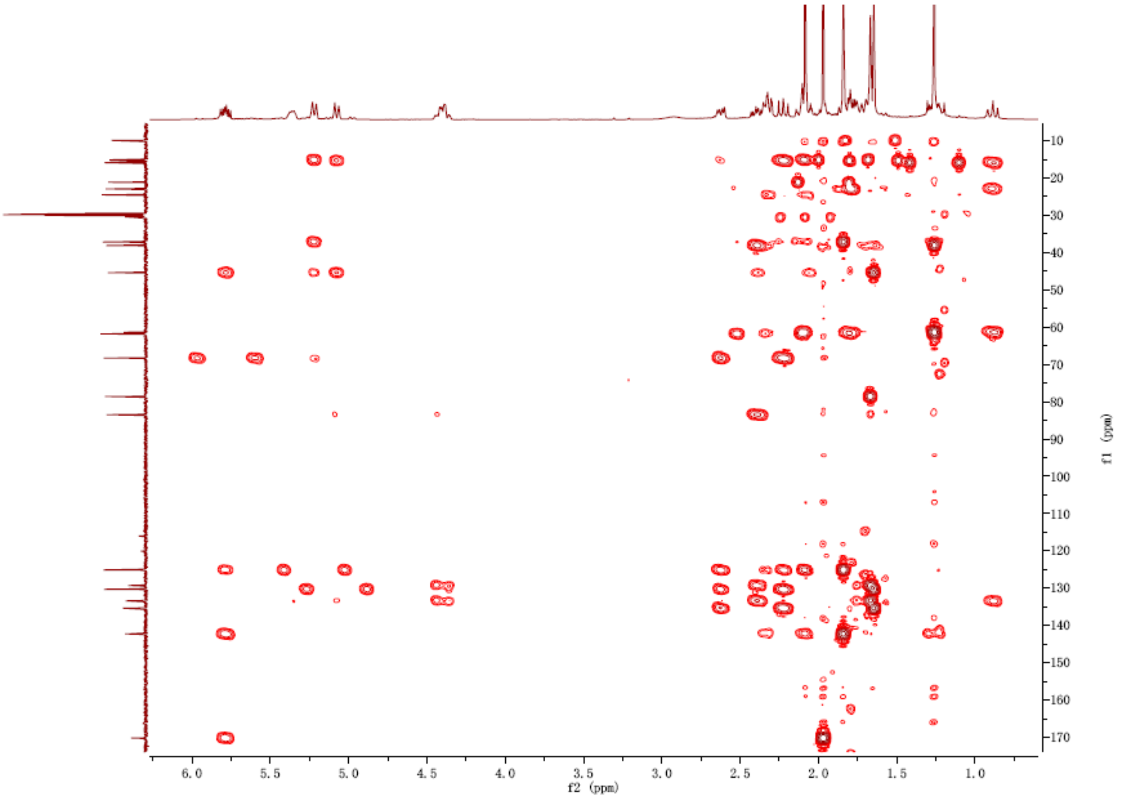

Figure S6. HMBC spectrum of compound **1**.

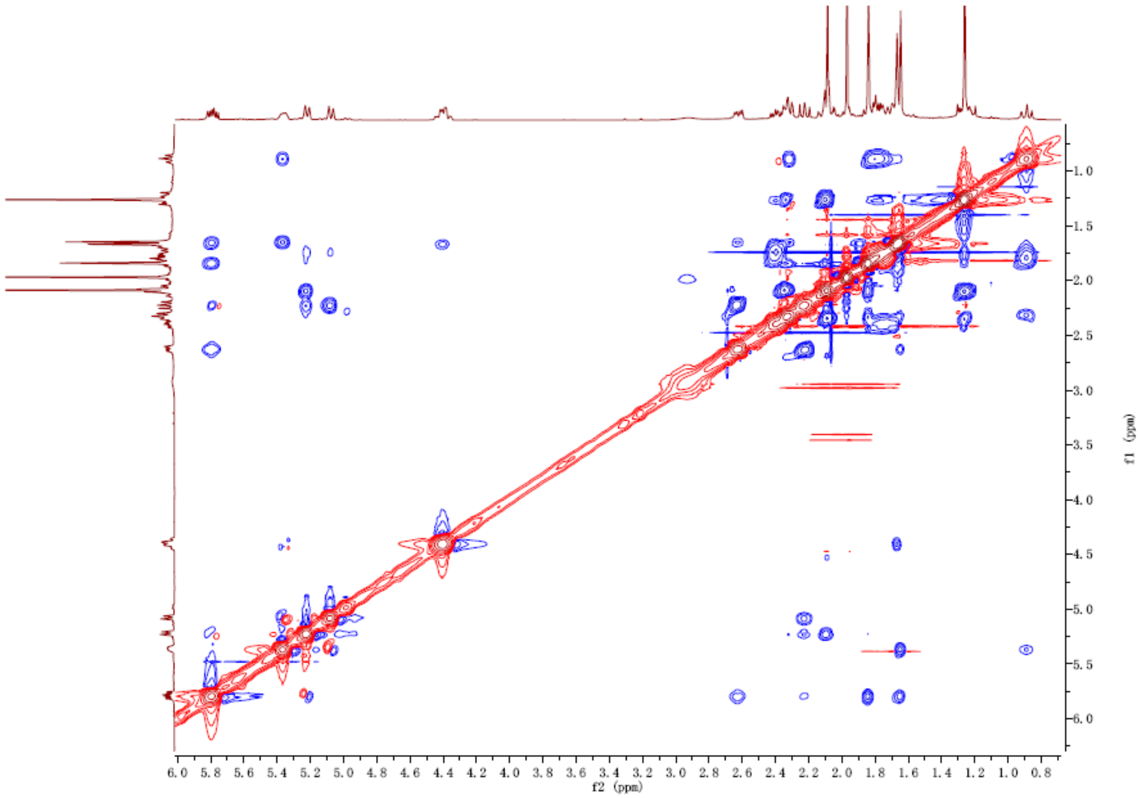

Figure S7. NOESY spectrum of compound **1**.

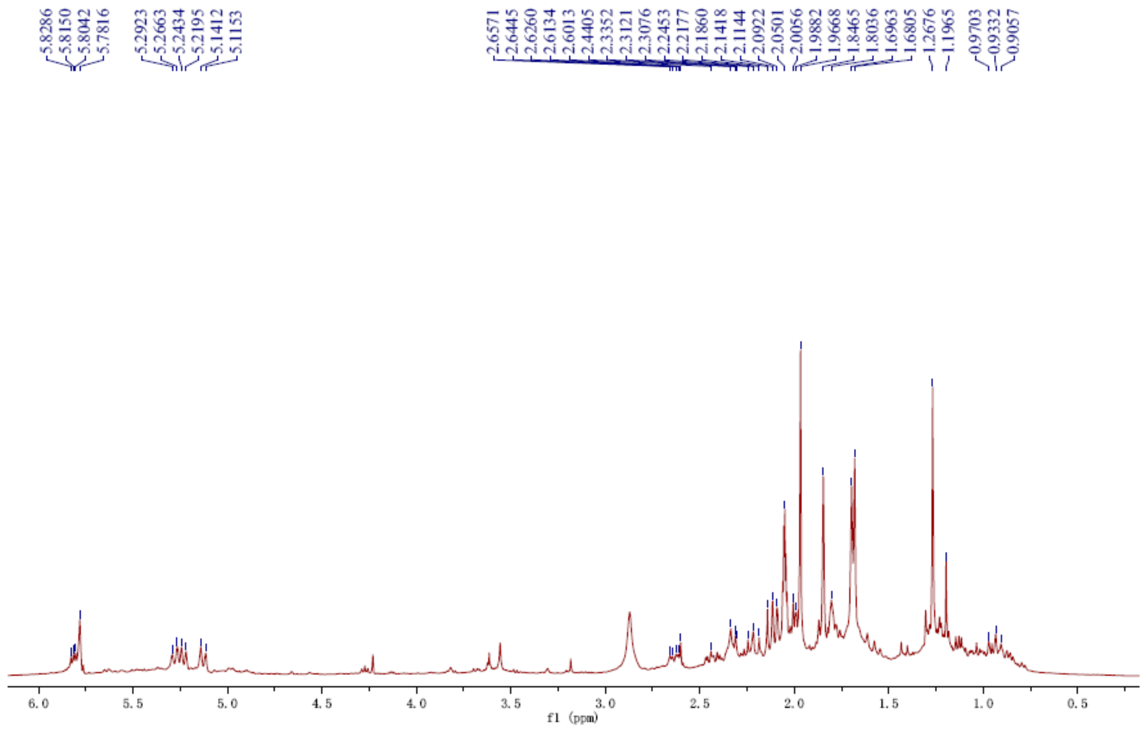

Figure S8. ^{1}H-NMR spectrum of compound **2**.

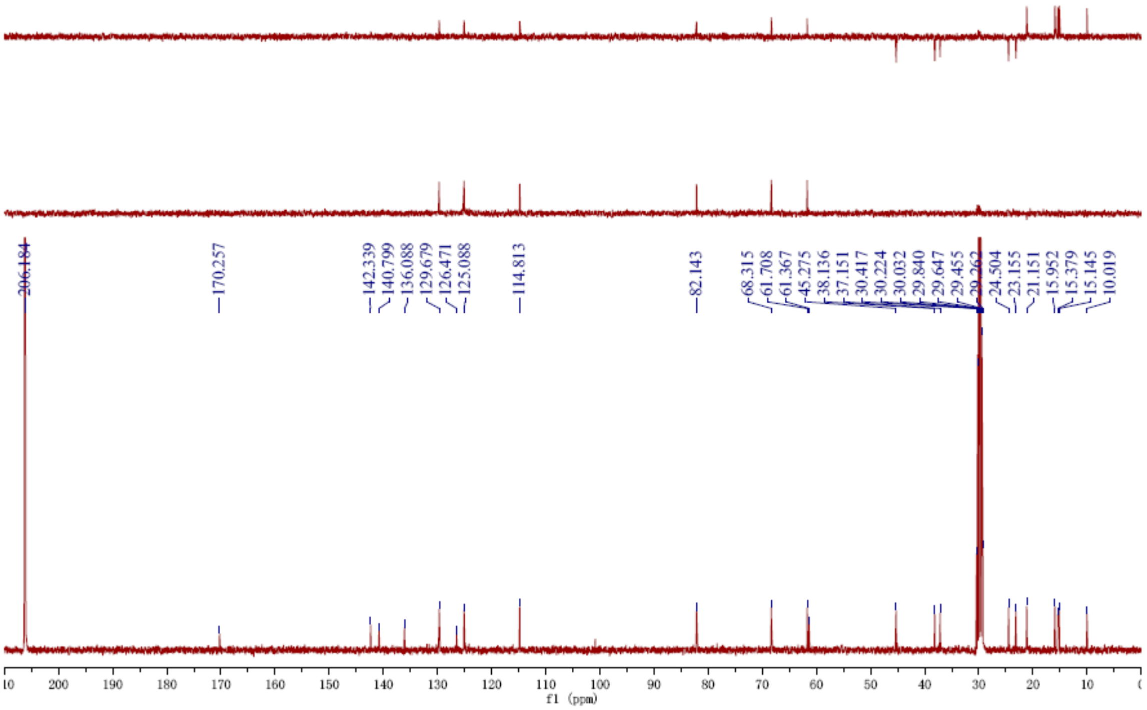

Figure S9. DEPT spectra of compound **2**.

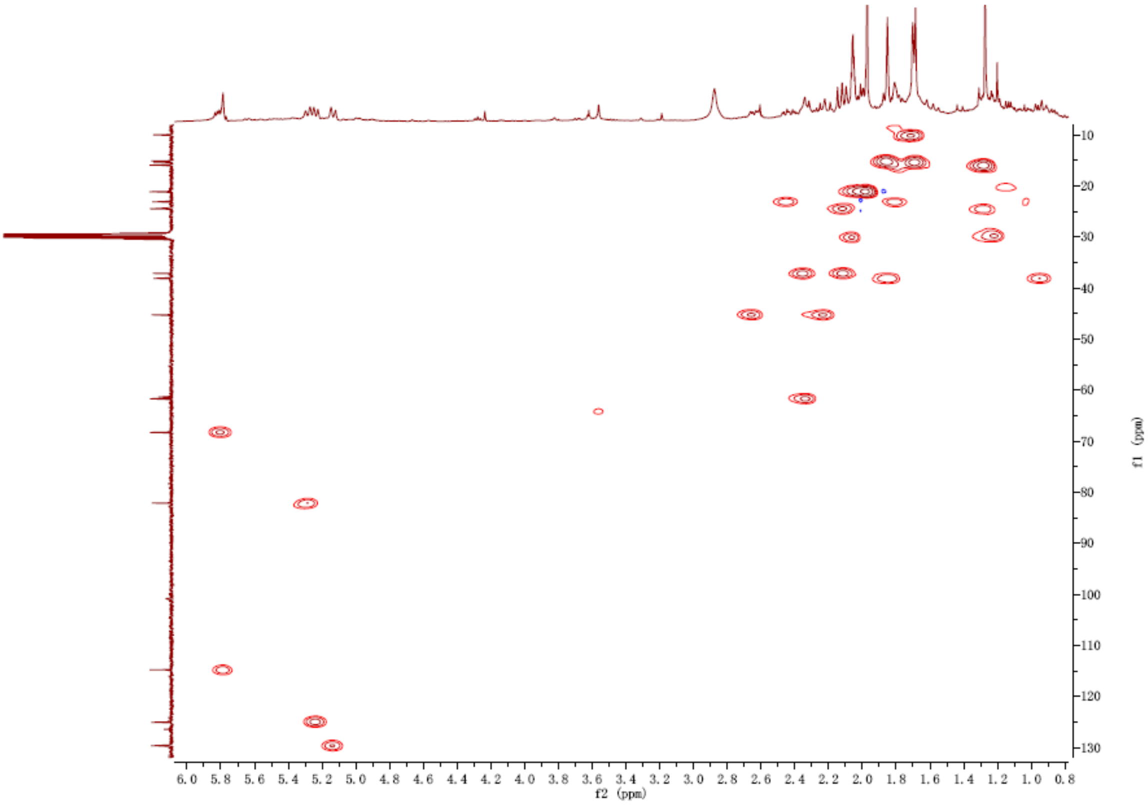

Figure S10. HSQC spectrum of compound **2**.

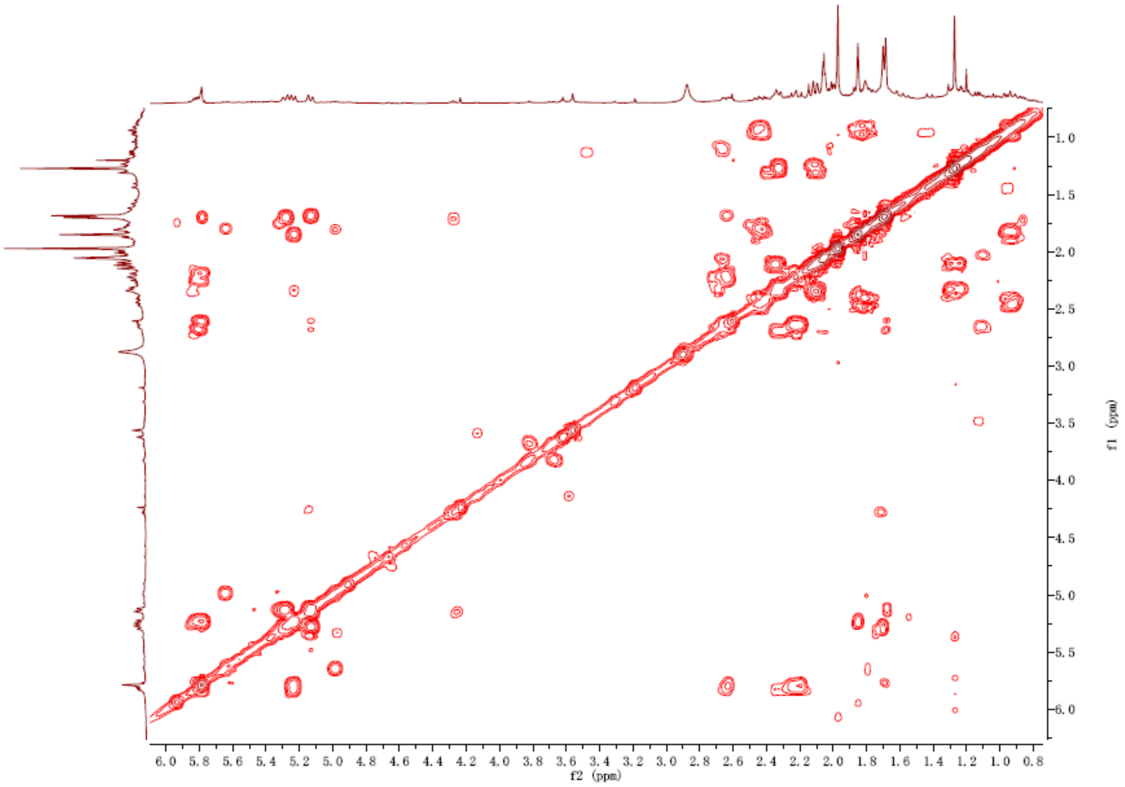

Figure S11. ^{1}H–^{1}H COSY spectrum of compound **2**.

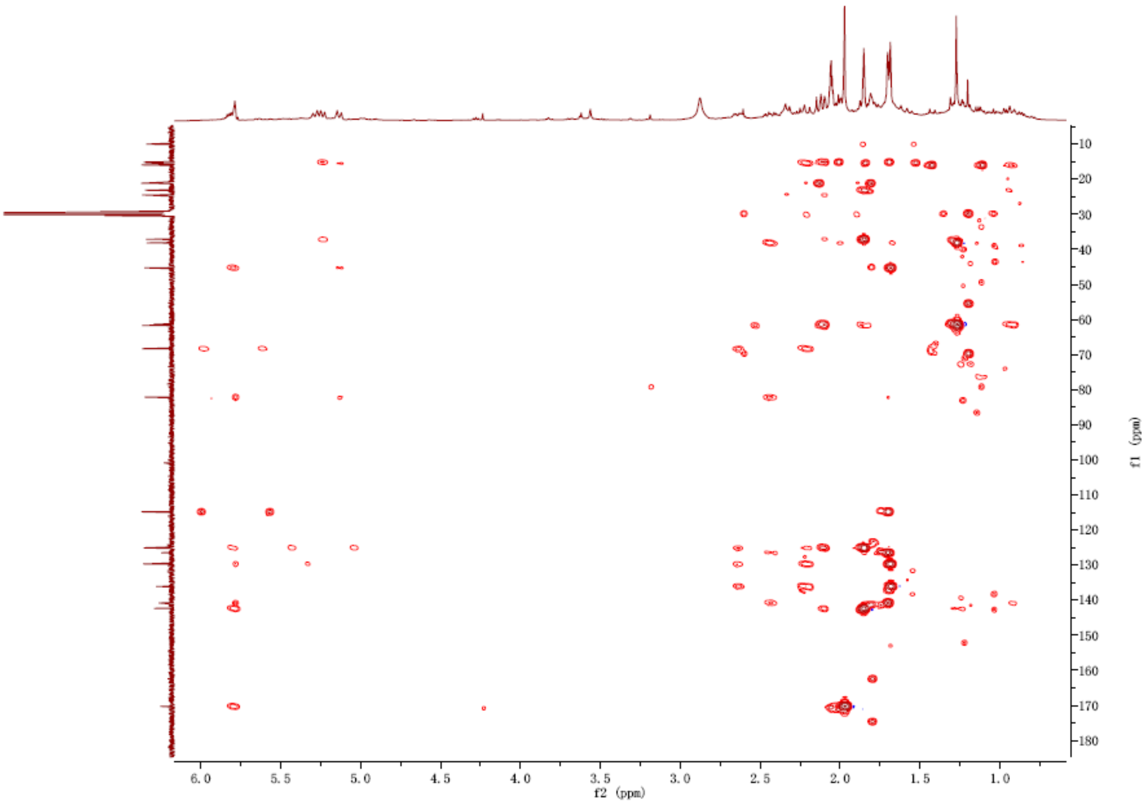

Figure S12. HMBC spectrum of compound **2**.

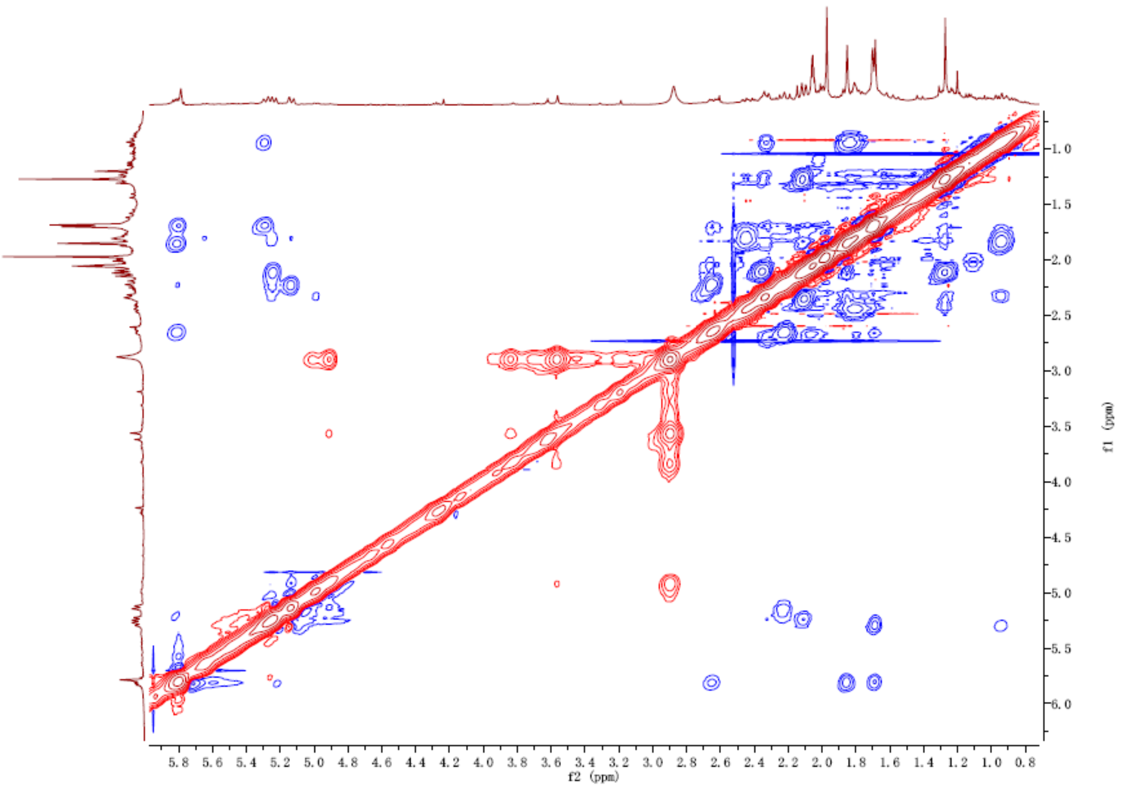

Figure S13. NOESY spectrum of compound **2**.

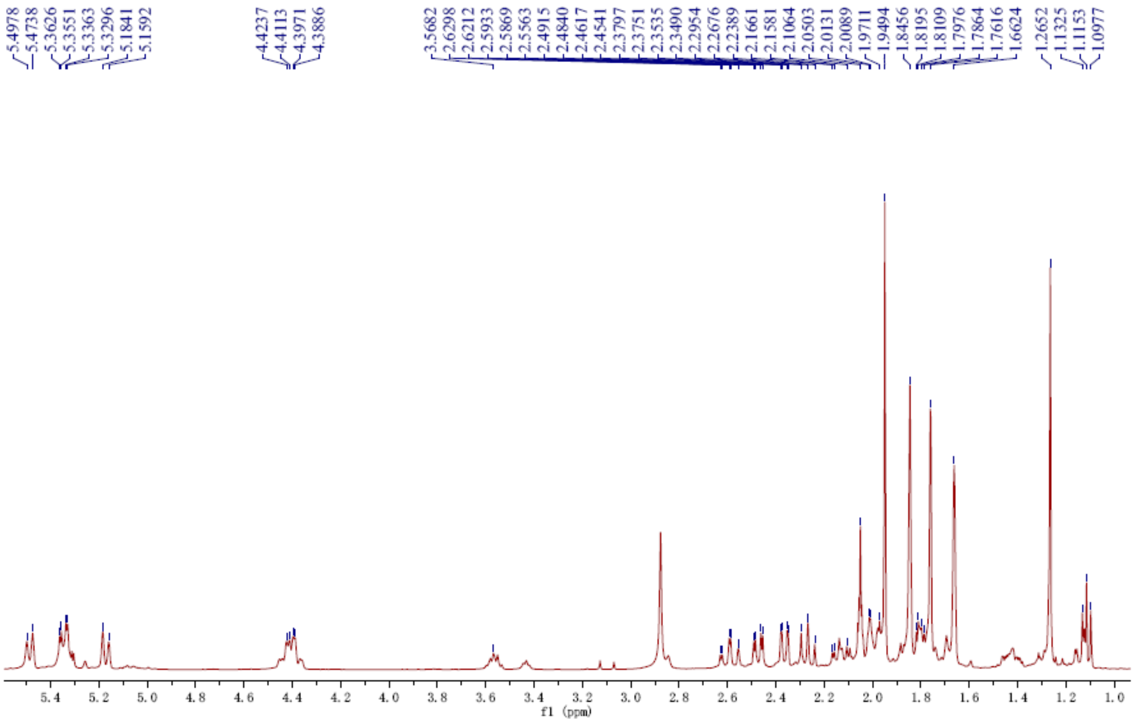

Figure S14. ^{1}H-NMR spectrum of compound **3**.

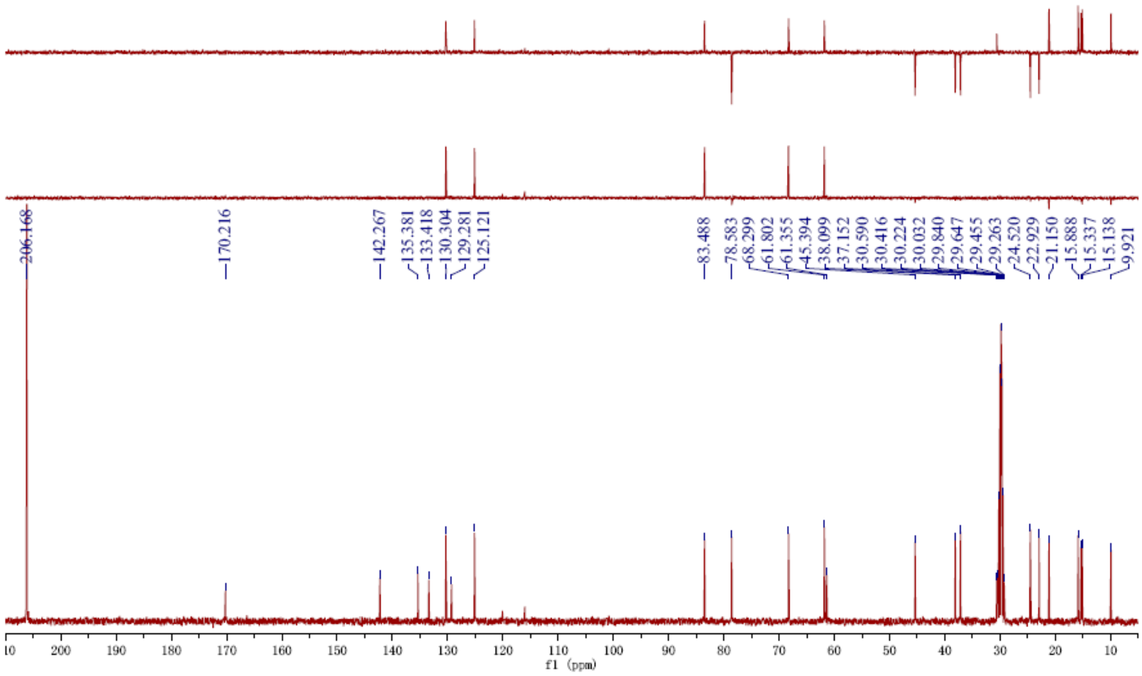

Figure S15. DEPT spectra of compound **3**.

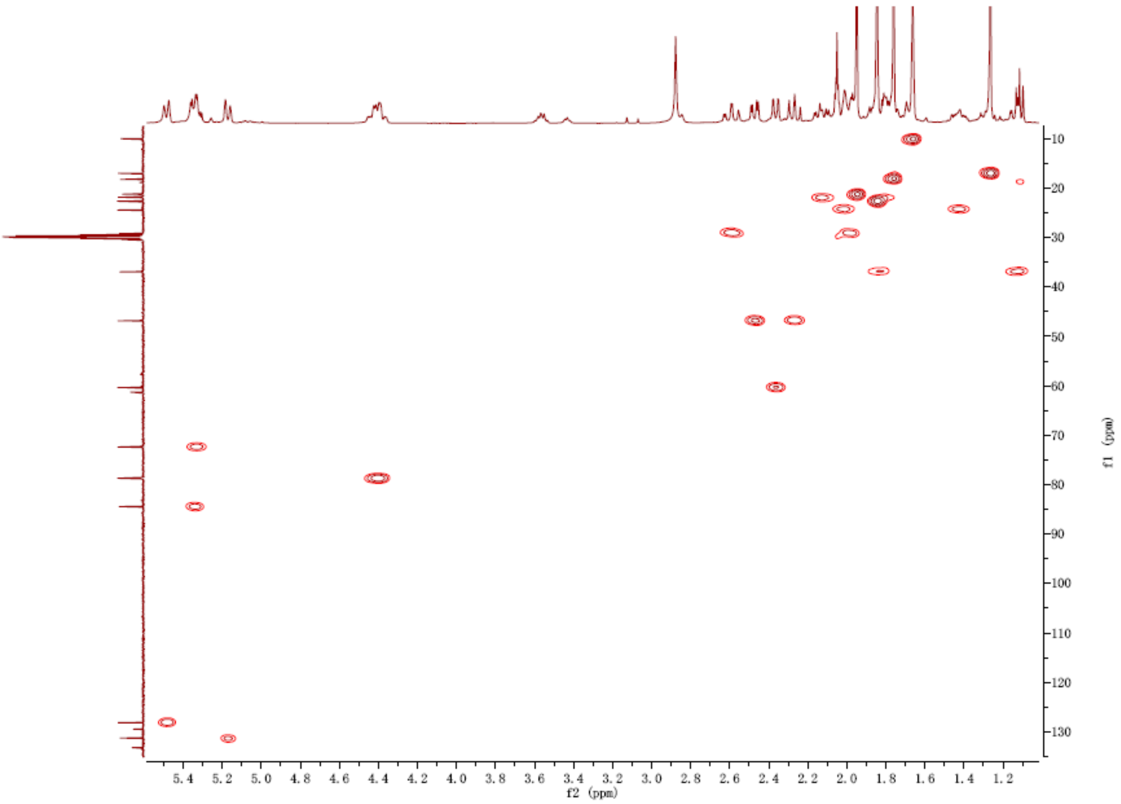

Figure S16. HSQC spectrum of compound **3**.

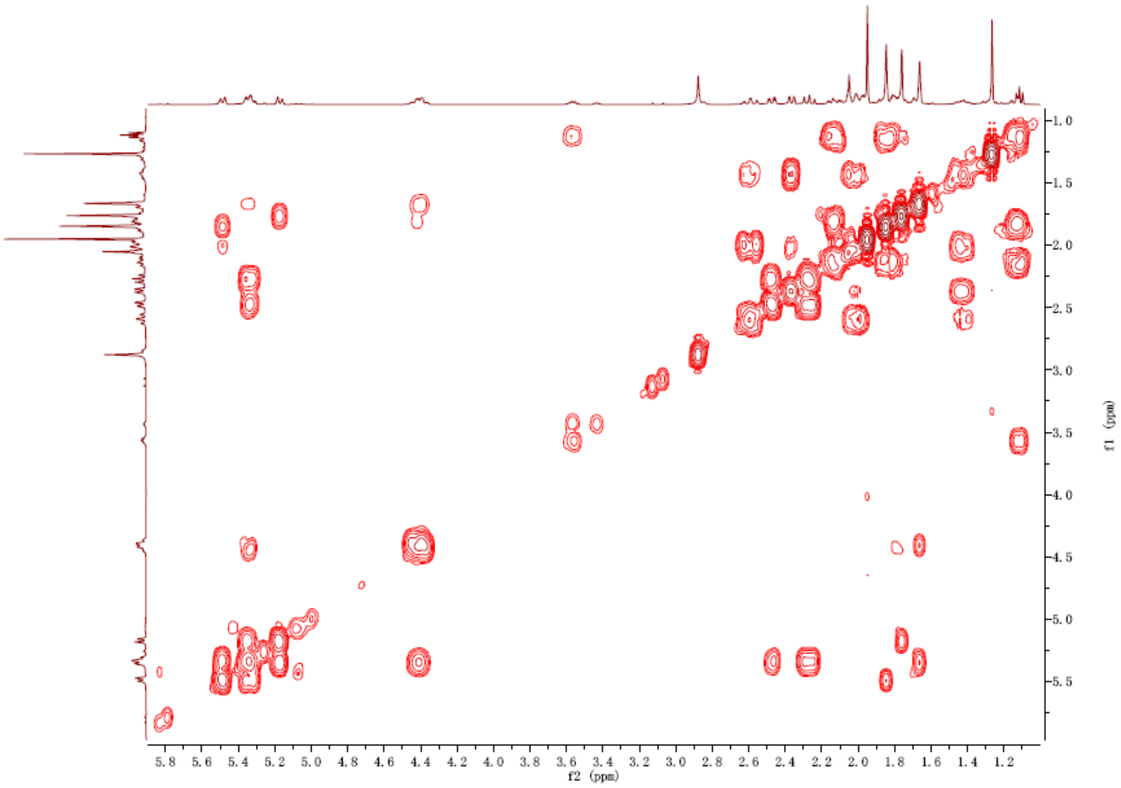

Figure S17. ¹H–¹H COSY spectrum of compound **3**.

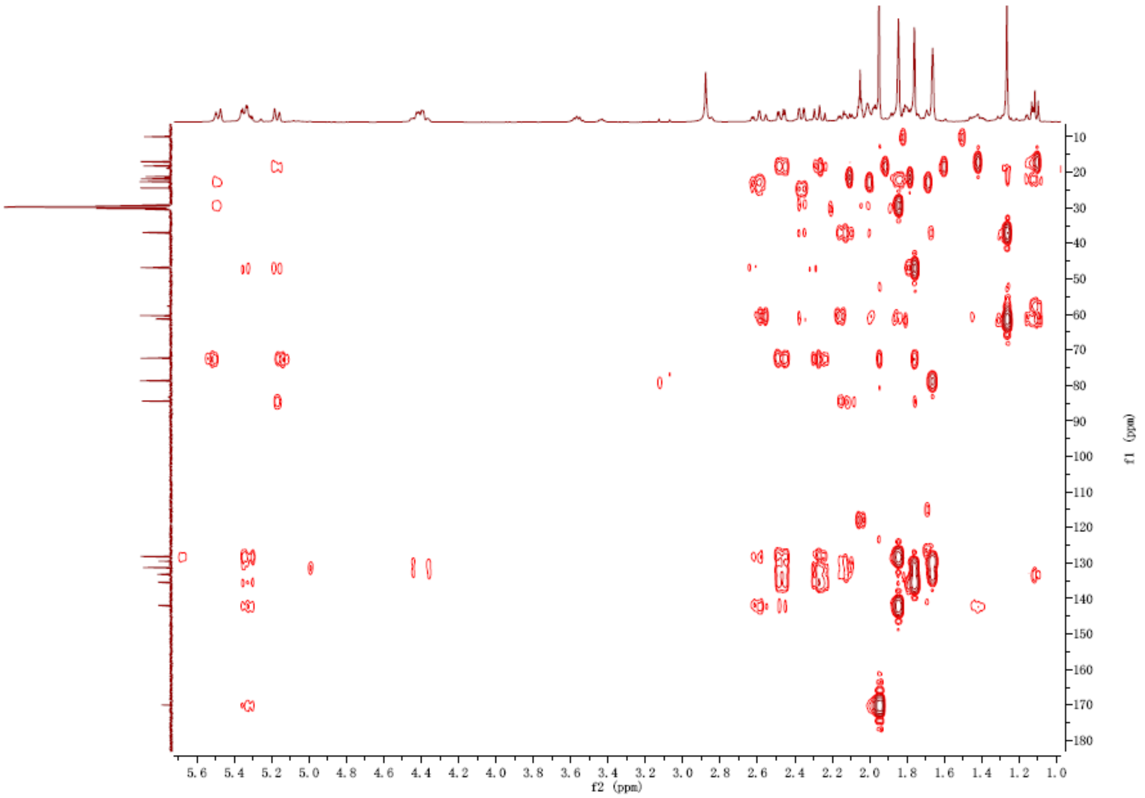

Figure S18. HMBC spectrum of compound **3**.

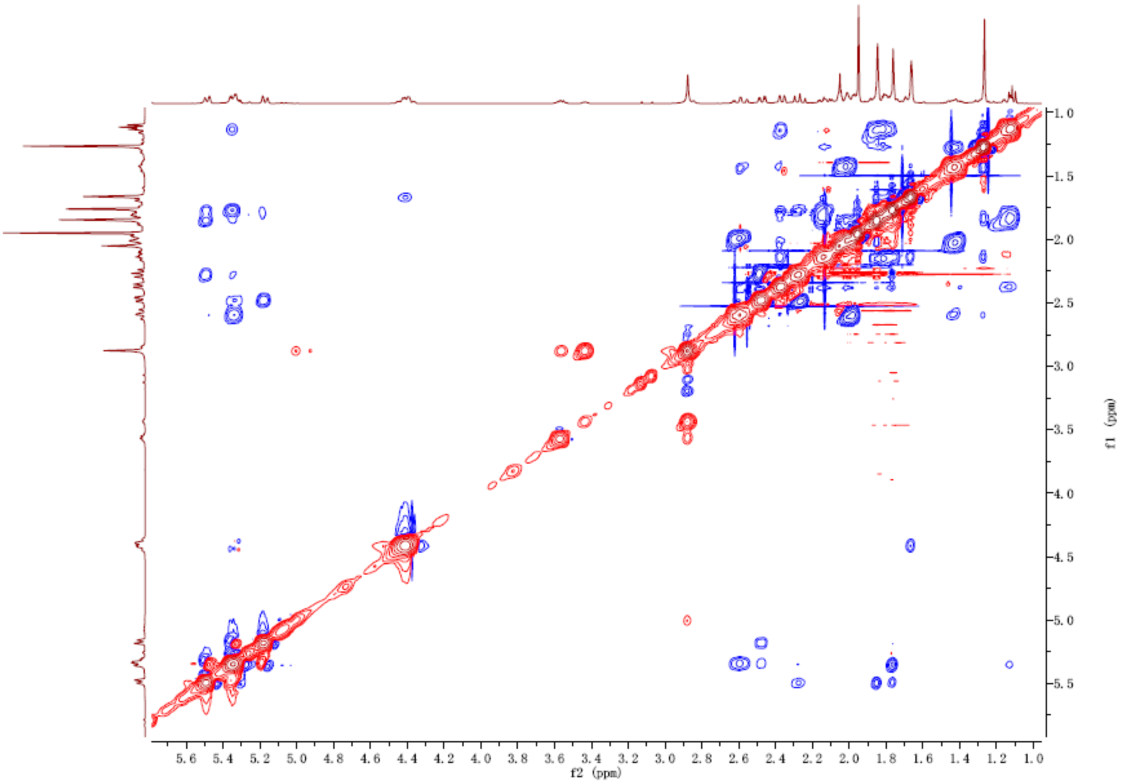

Figure S19. NOESY spectrum of compound **3**.

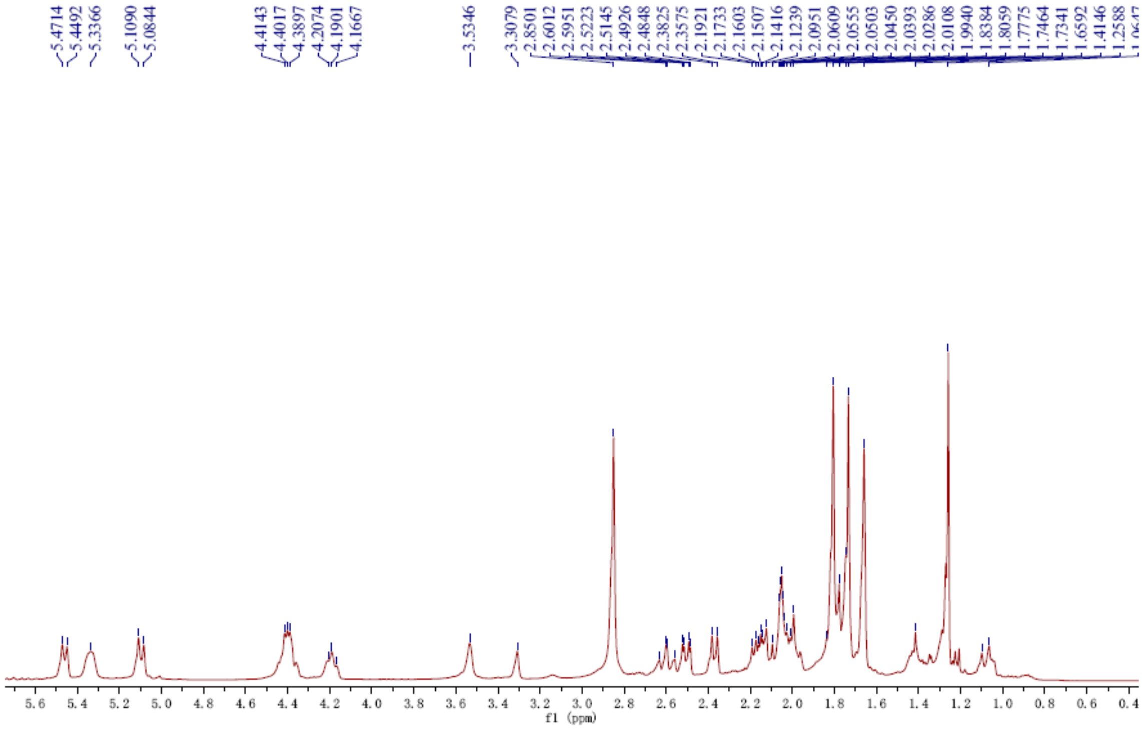

Figure S20. ^{1}H-NMR spectrum of compound **4**.

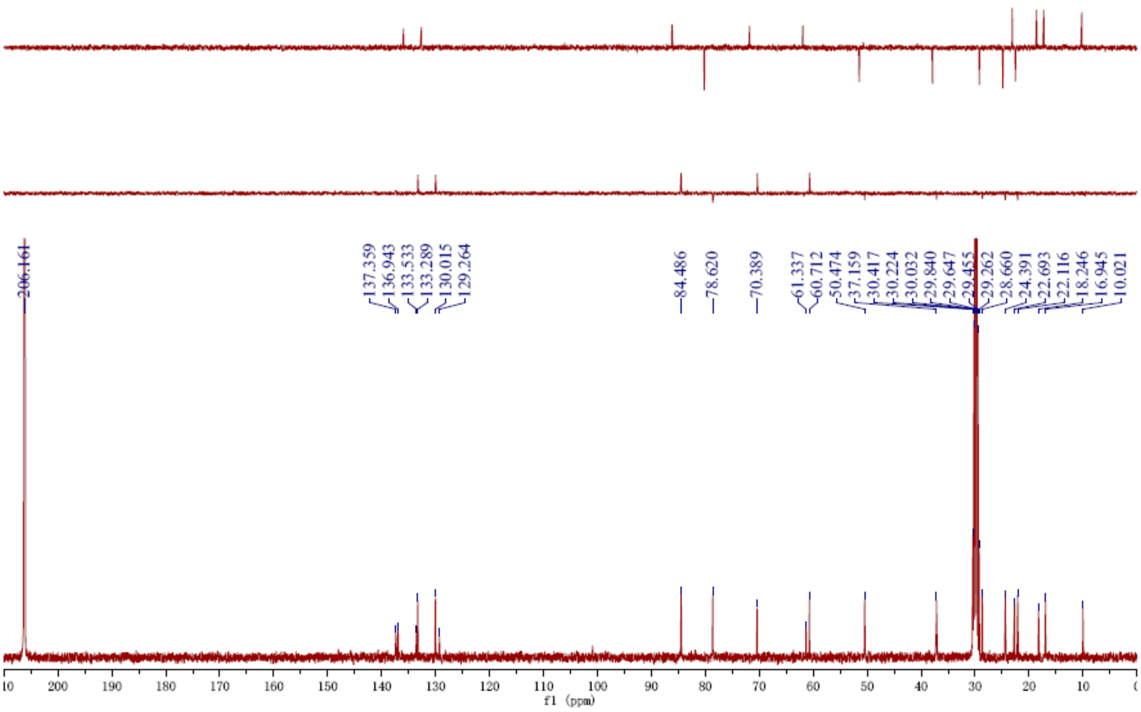

Figure S21. DEPT spectra of compound **4**.

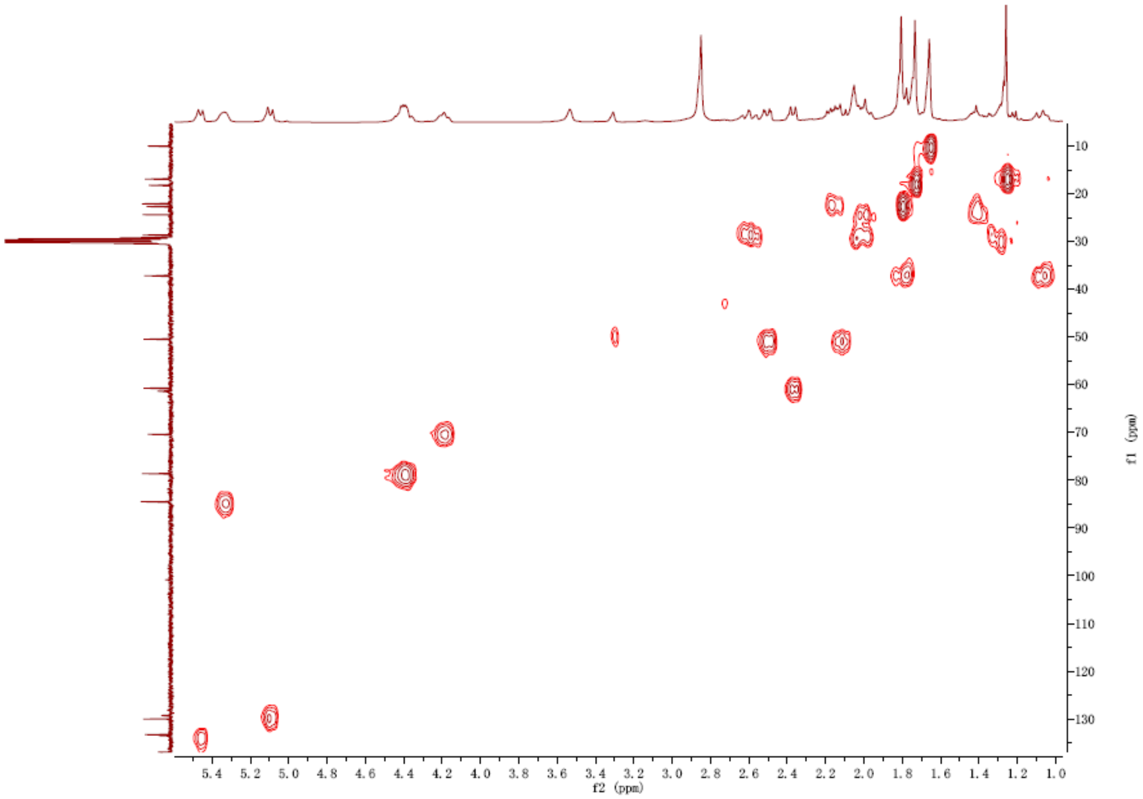

Figure S22. HSQC spectrum of compound **4**.

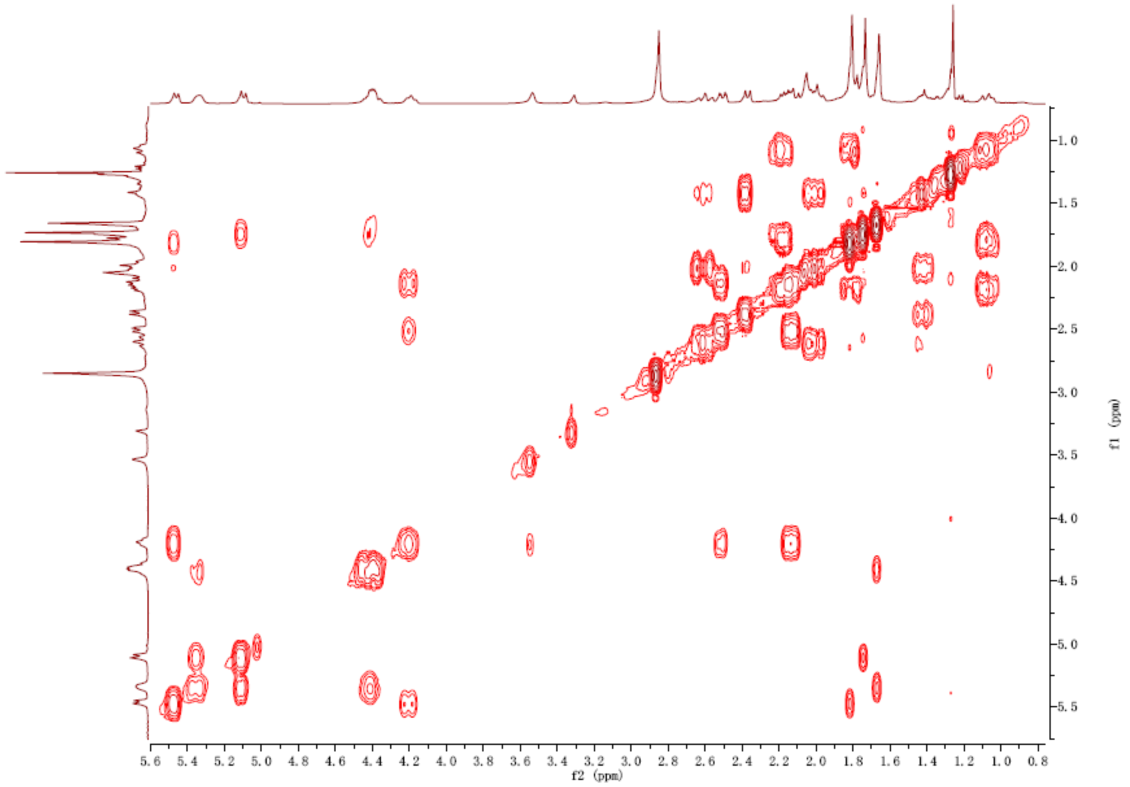

Figure S23. ^{1}H–^{1}H COSY spectrum of compound **4**.

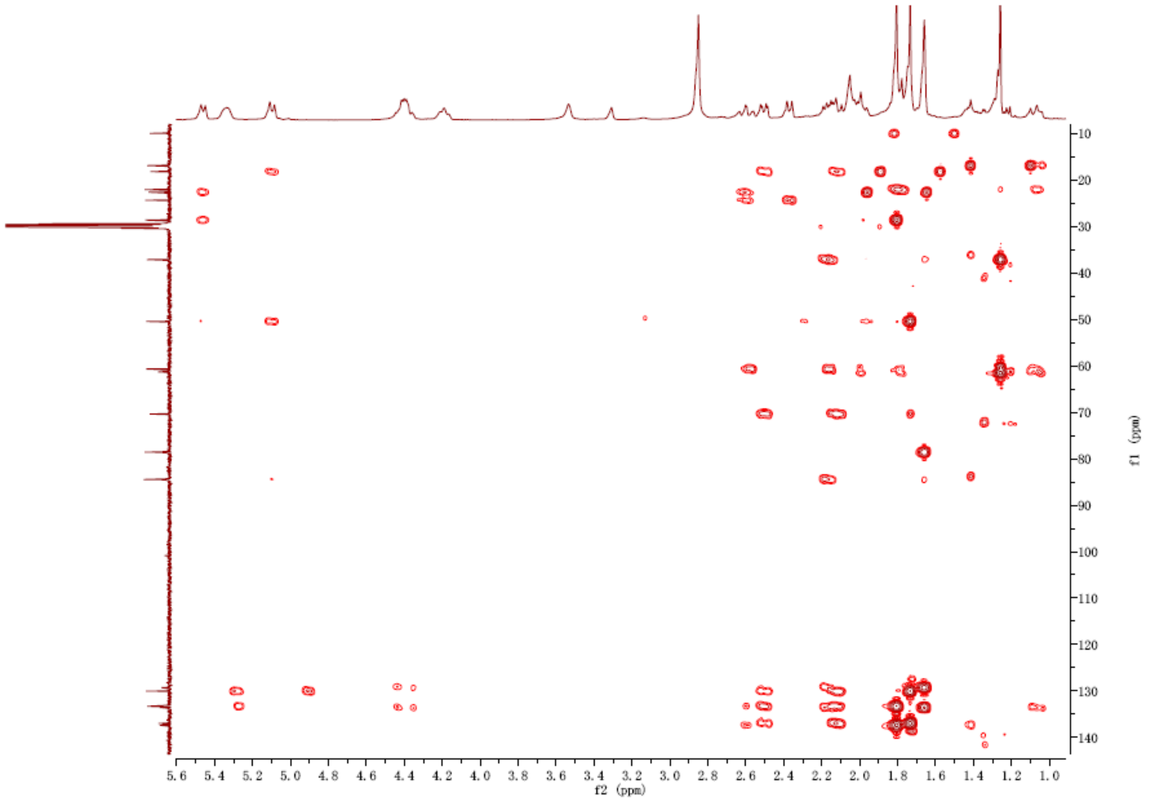

Figure S24. HMBC spectrum of compound **4**.

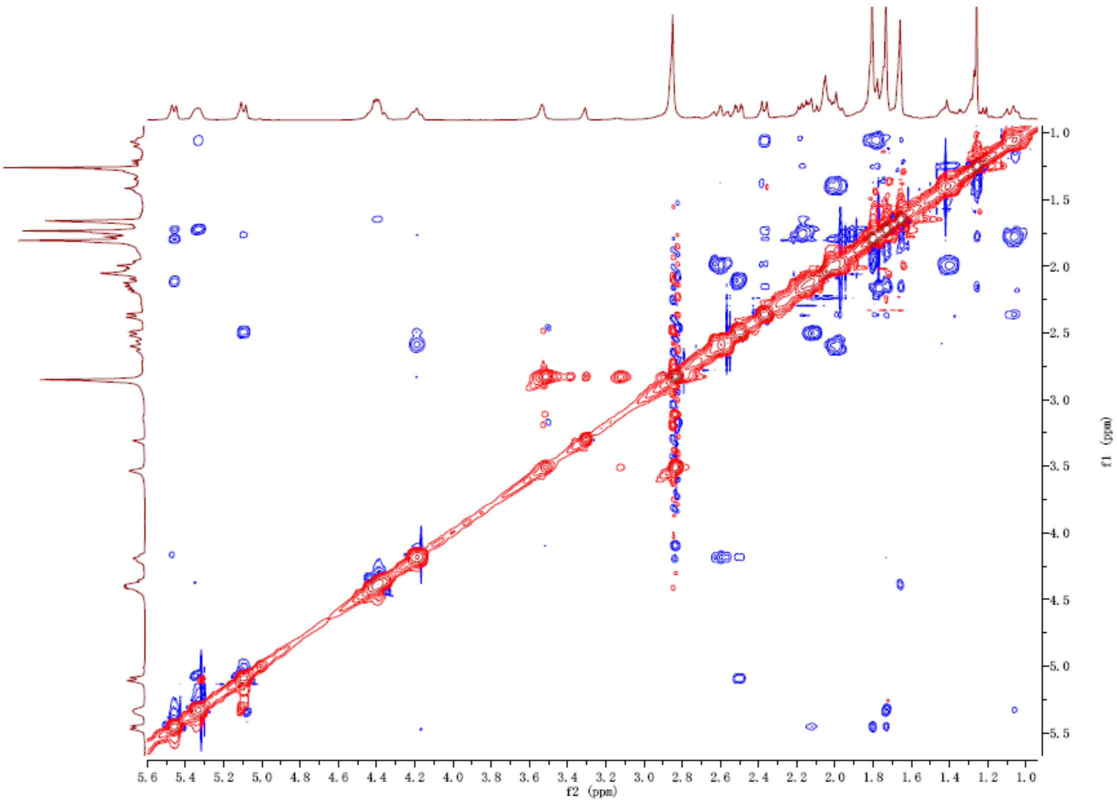

Figure S25. NOESY spectrum of compound **4**.

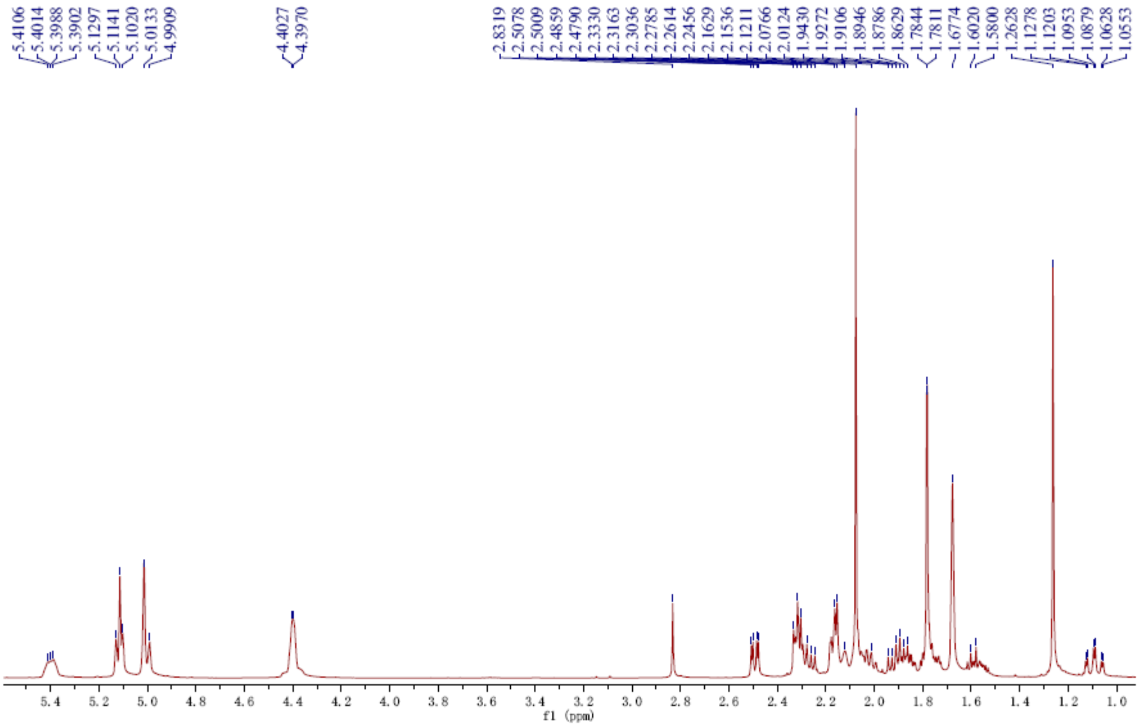

Figure S26. ^{1}H-NMR spectrum of compound **5**.

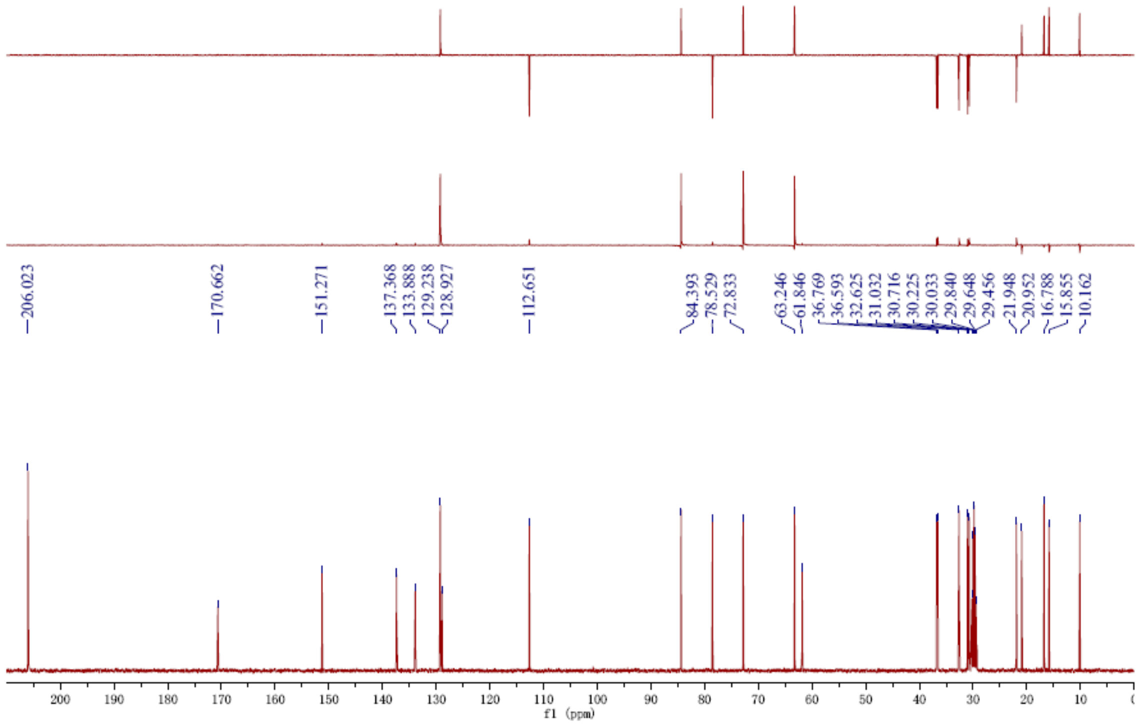

Figure S27. DEPT spectra of compound **5**.

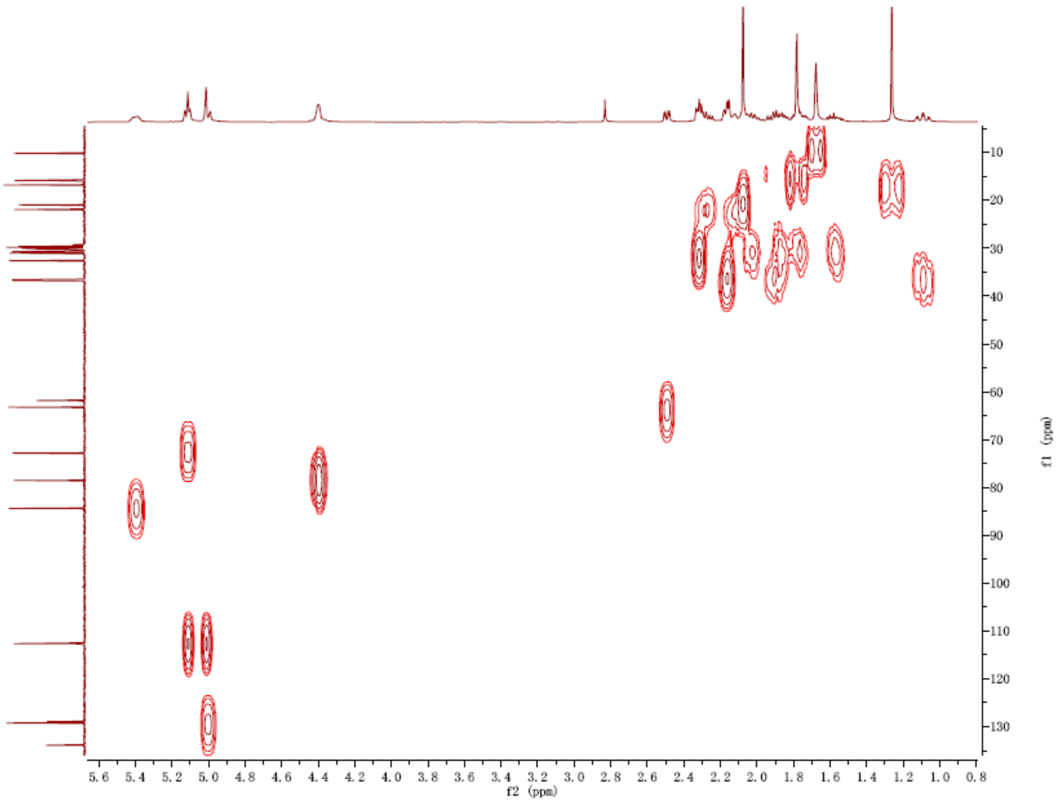

Figure S28. HSQC spectrum of compound **5**.

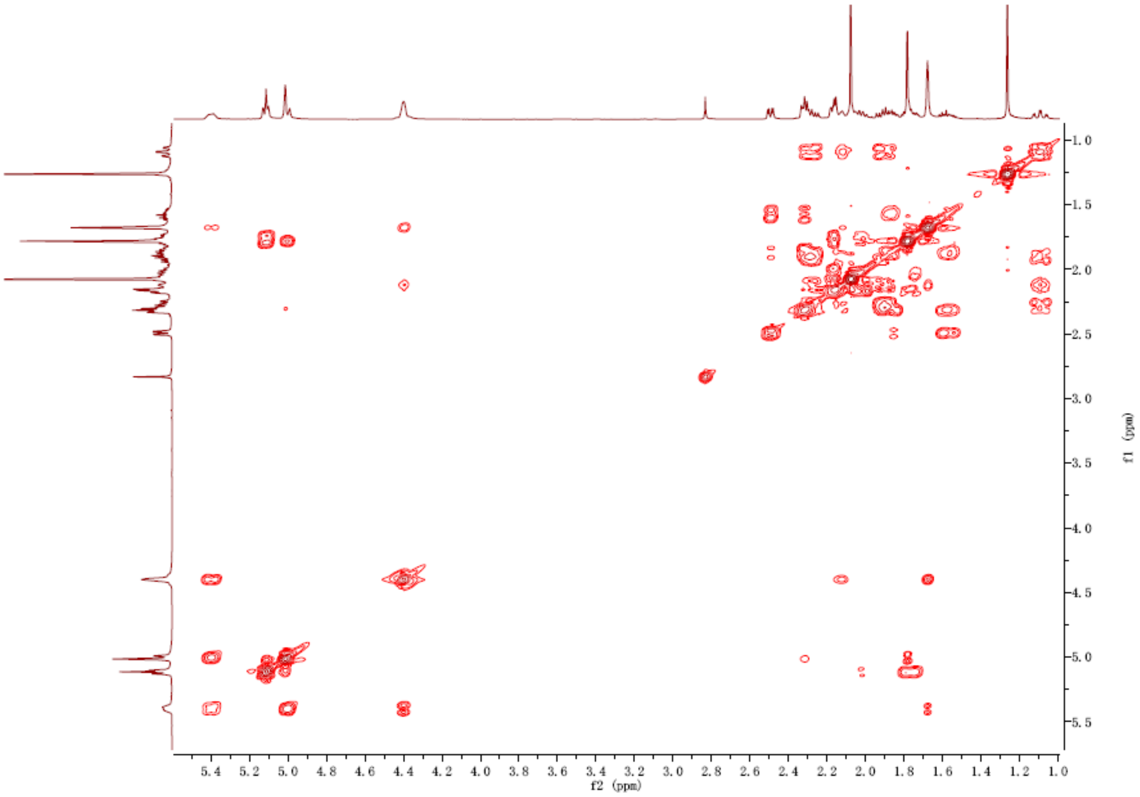

Figure S29. ^{1}H–^{1}H COSY spectrum of compound **5**.

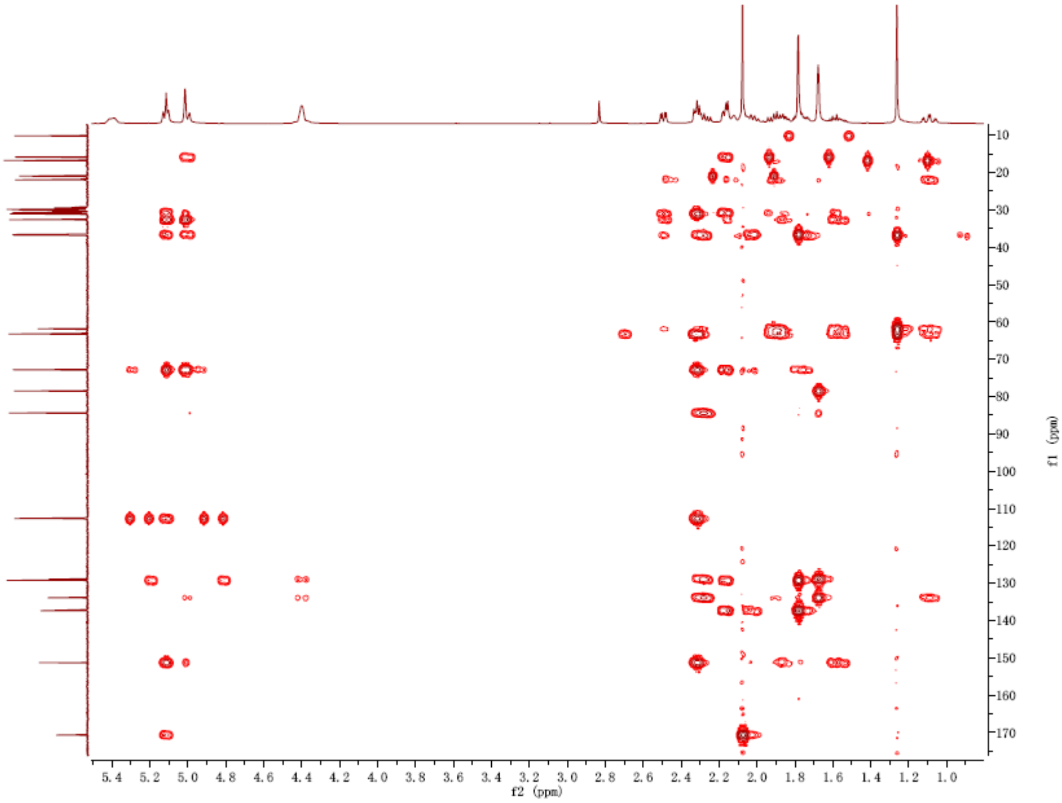

Figure S30. HMBC spectrum of compound **5**.

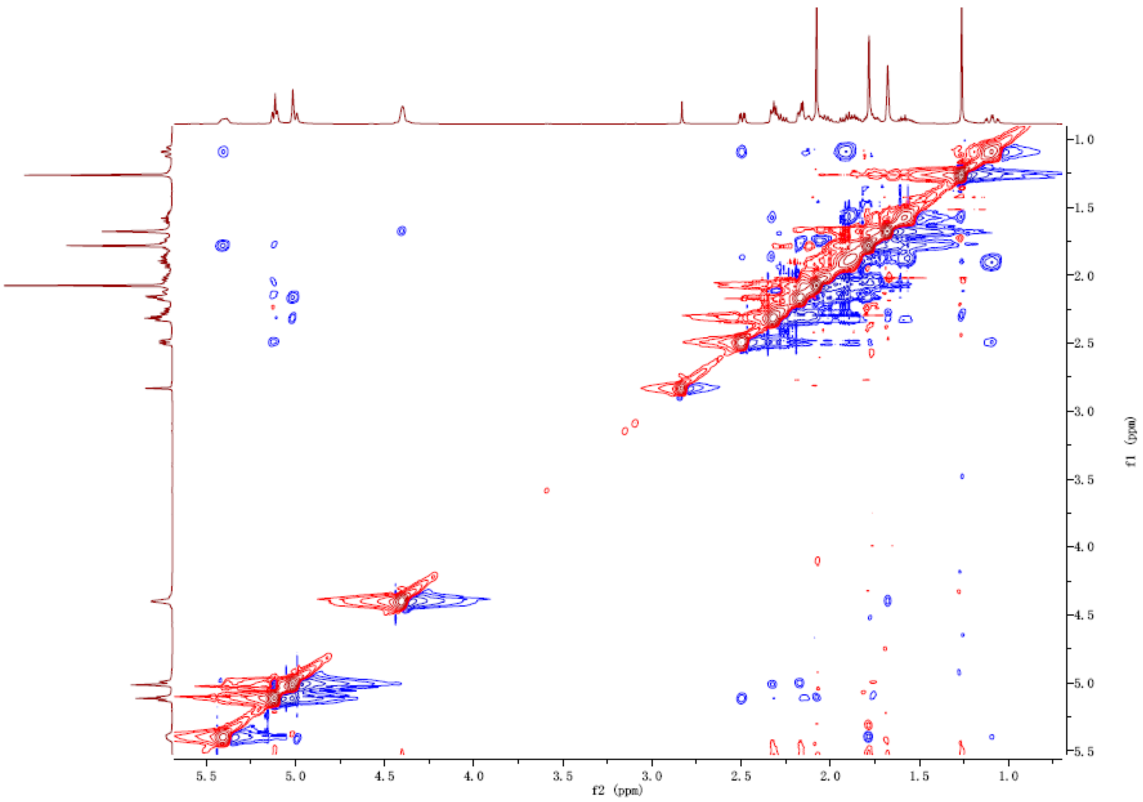

Figure S31. NOESY spectrum of compound **5**.

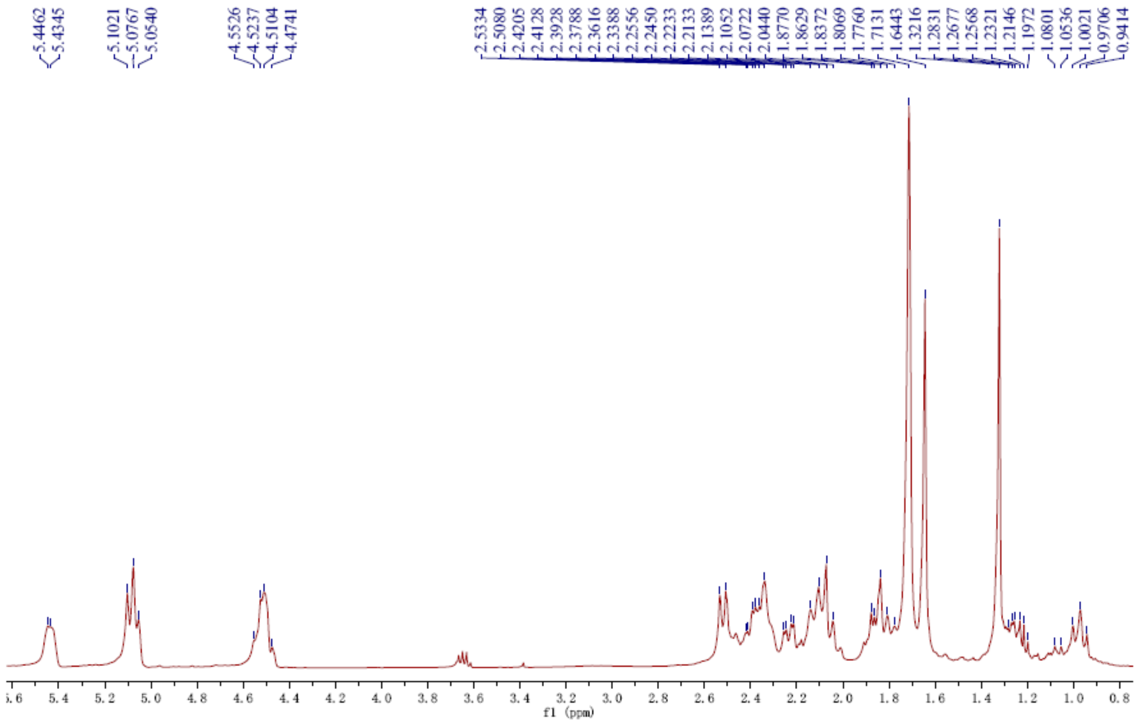

Figure S32. ^{1}H-NMR spectrum of compound **6**.

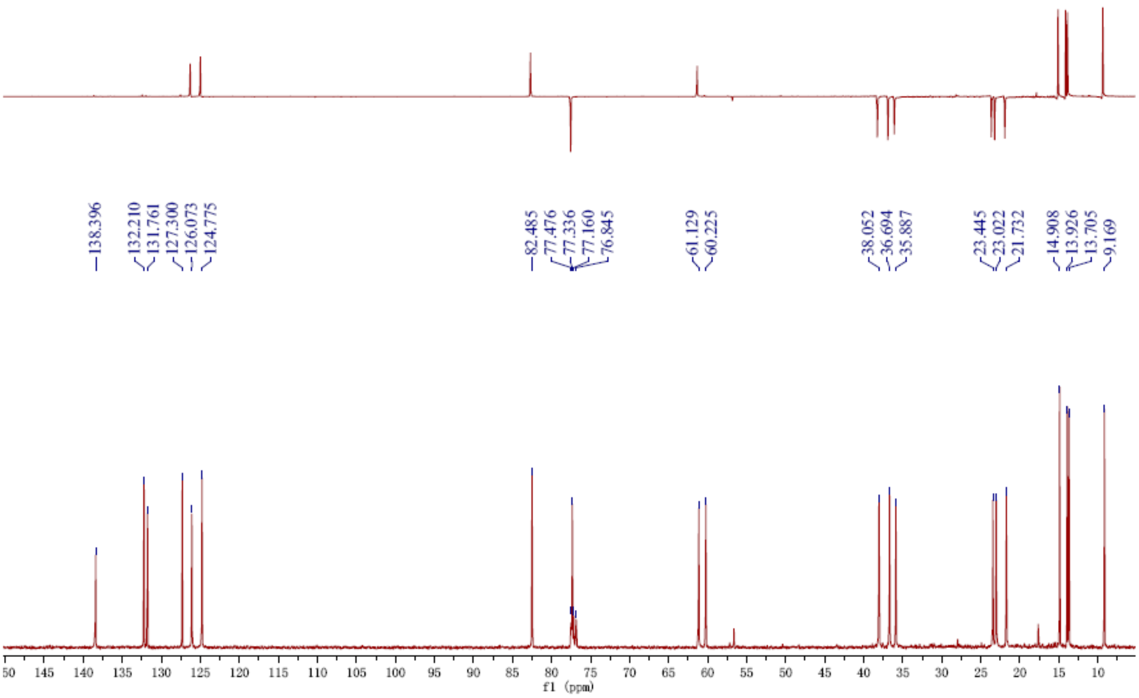

Figure S33. DEPT spectra of compound **6**.

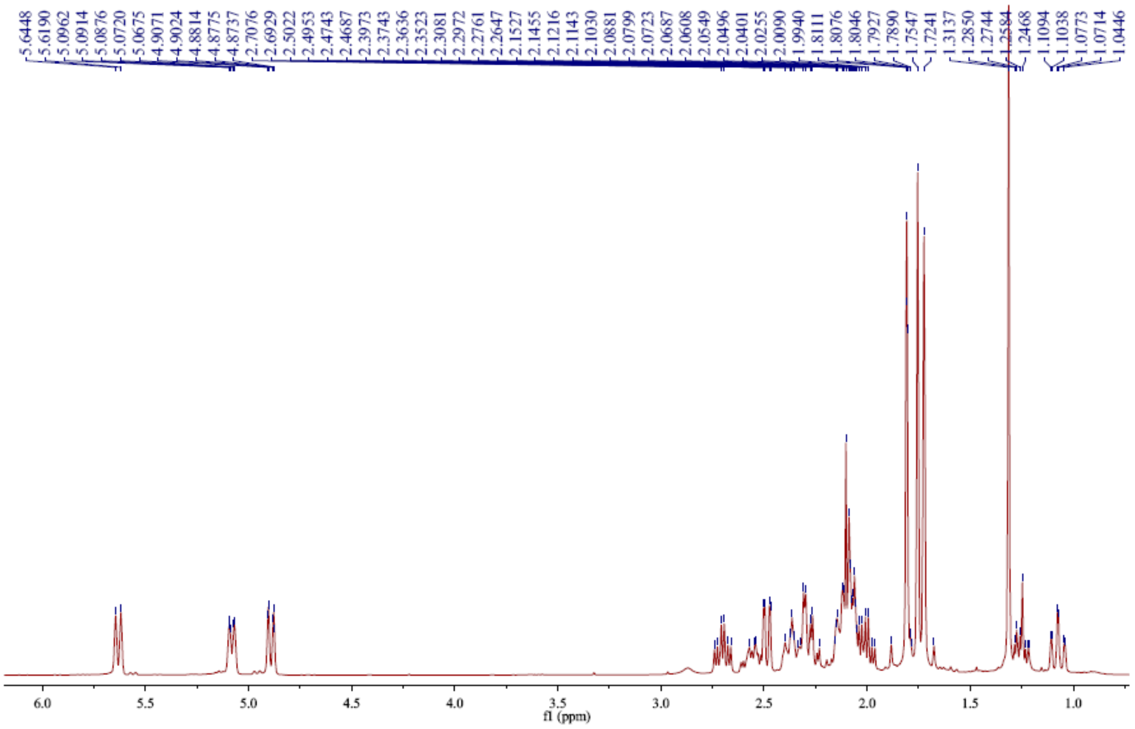

Figure S34. ^{1}H-NMR spectrum of compound **7**.

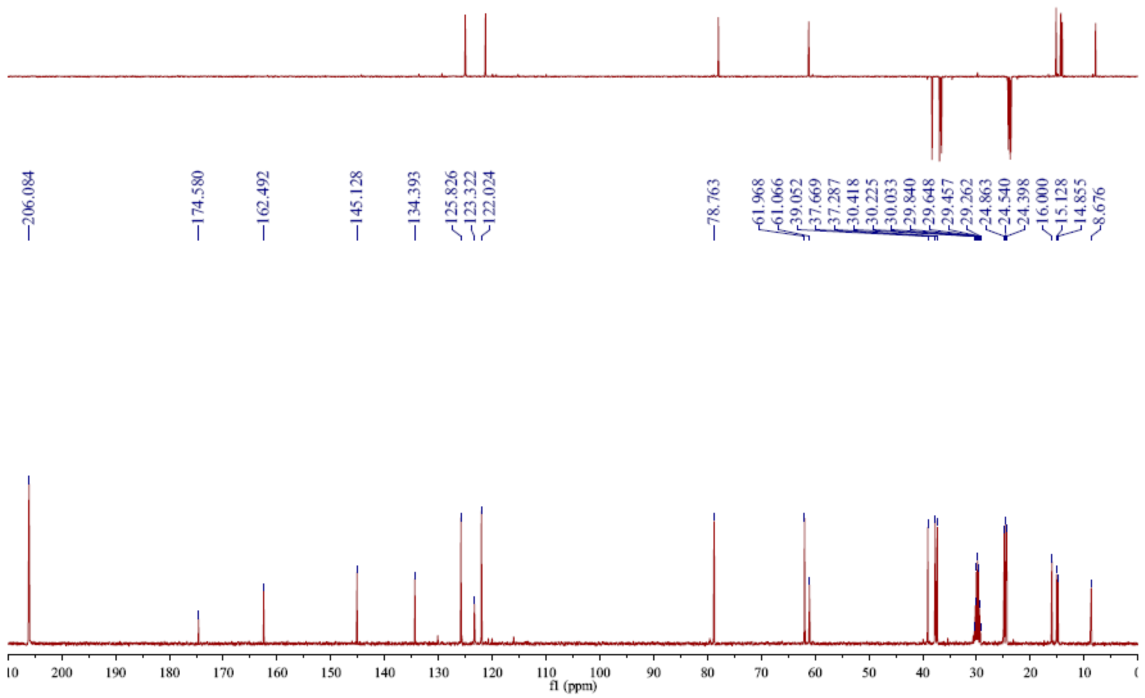

Figure S35. DEPT spectra of compound **7**.

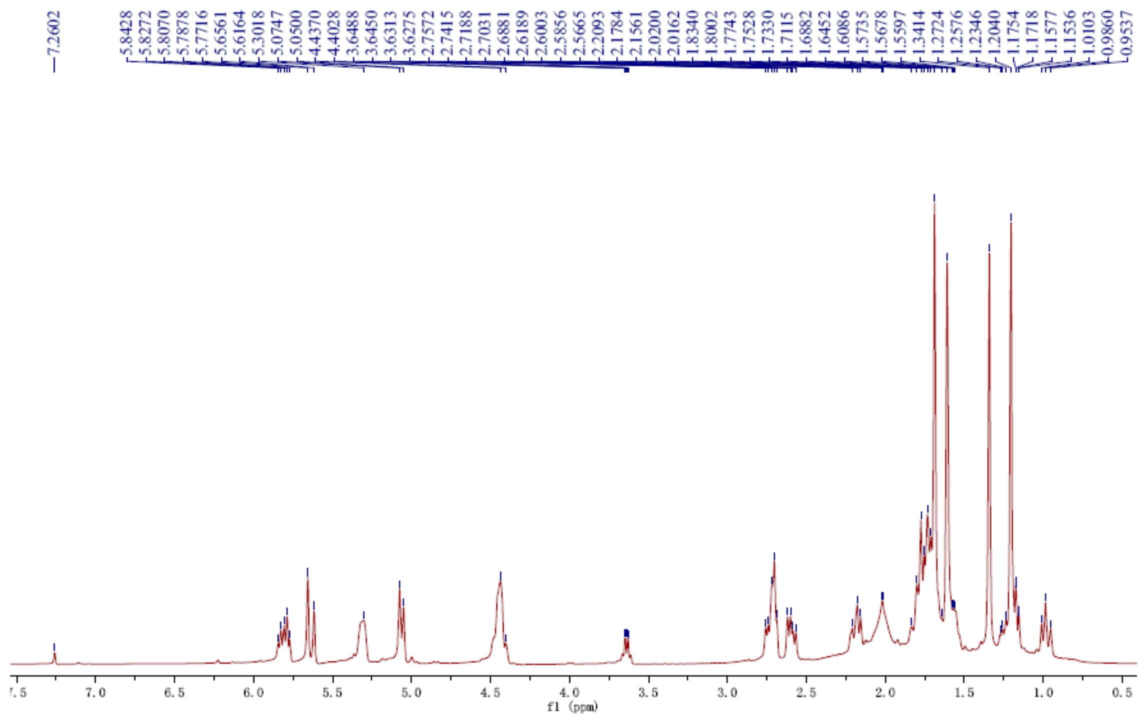

Figure S36. ^{1}H-NMR spectrum of compound **8**.

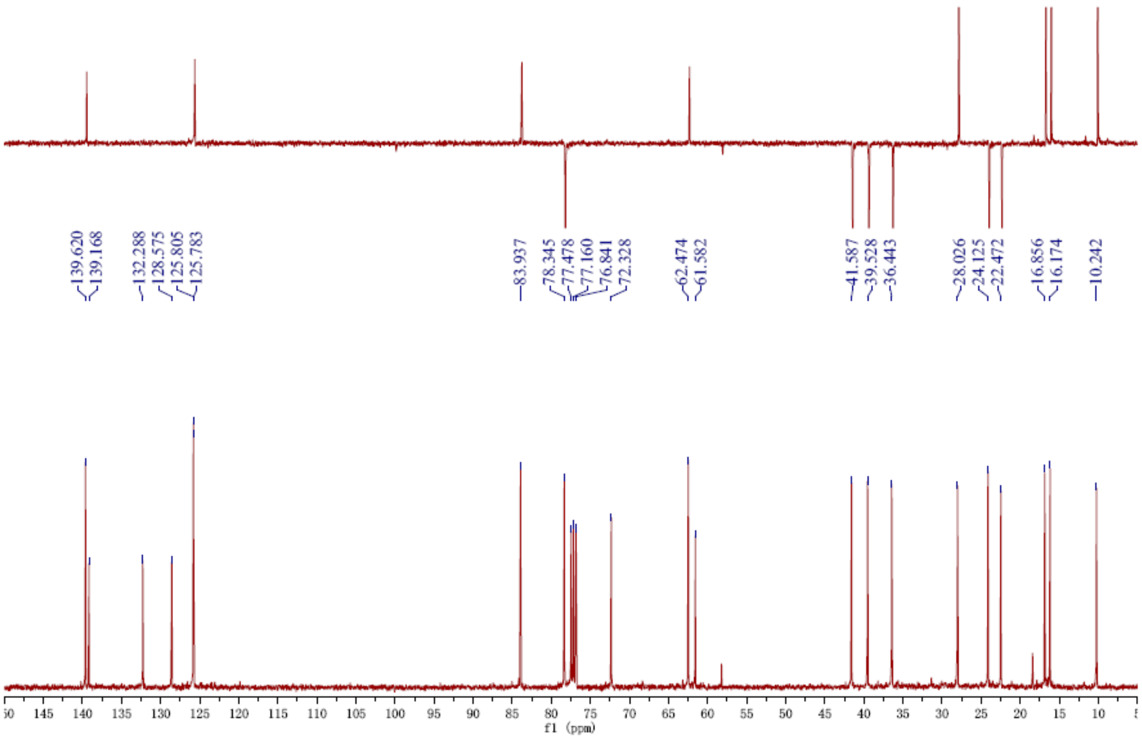

Figure S37. DEPT spectra of compound **8**.
